# Autoinducer-2-mediated communication network within human gut microbiota

**DOI:** 10.1093/ismejo/wraf204

**Published:** 2025-09-11

**Authors:** Qingying Fan, Hengxi Sun, Xueyuan Lin, Wenguang Yang, Xihui Shen, Lei Zhang

**Affiliations:** State Key Laboratory for Crop Stress Resistance and High-Efficiency Production, Shaanxi Key Laboratory of Agricultural and Environmental Microbiology, College of Life Sciences, Northwest A&F University, Yangling, Shaanxi 712100, China; MOE Key Laboratory of Contemporary Anthropology, Department of Anthropology and Human Genetics, School of Life Sciences, Fudan University, Shanghai 200438, China; State Key Laboratory for Crop Stress Resistance and High-Efficiency Production, Shaanxi Key Laboratory of Agricultural and Environmental Microbiology, College of Life Sciences, Northwest A&F University, Yangling, Shaanxi 712100, China; State Key Laboratory for Crop Stress Resistance and High-Efficiency Production, Shaanxi Key Laboratory of Agricultural and Environmental Microbiology, College of Life Sciences, Northwest A&F University, Yangling, Shaanxi 712100, China; State Key Laboratory for Crop Stress Resistance and High-Efficiency Production, Shaanxi Key Laboratory of Agricultural and Environmental Microbiology, College of Life Sciences, Northwest A&F University, Yangling, Shaanxi 712100, China; State Key Laboratory for Crop Stress Resistance and High-Efficiency Production, Shaanxi Key Laboratory of Agricultural and Environmental Microbiology, College of Life Sciences, Northwest A&F University, Yangling, Shaanxi 712100, China

**Keywords:** Quorum sensing, autoinducer-2, human gut microbiota, AI-2 receptors, communication network

## Abstract

Quorum sensing (QS) is a chemical communication process that connects microbial members in various microbial systems. Bacterial communication networks mediated by QS play important roles in the regulation of intestinal microecological balance as well as nutrition and metabolism of the host. However, how human gut microbes utilize QS signals to communicate with one another remains largely unknown. In this study, we first examined the prevalence and abundance of genes encoding QS signal synthases in 3329 species representatives clustered from 289232 prokaryotic genomes in the Unified Human Gastrointestinal Genome collection. Our results show autoinducer-2 (AI-2) is the most prevalent QS signal within the human gut microbiota, with the synthase gene *luxS* being found in 2039 species mainly distributed within *Firmicutes*, *Actinobacteriota*, *Bacteroidota*, and *Proteobacteria*. Furthermore, 299 species carry genes encoding one or more types of AI-2 receptors (LuxP-, LsrB-, dCache_1-, and GAPES1-type). The dCache_1- and GAPES1-type receptors can function as methyl-accepting chemotaxis proteins, histidine kinases, c-di-GMP synthases and/or c-di-GMP-specific phosphodiesterases, serine phosphatases, and serine/threonine kinases, suggesting the diversity of AI-2-mediated interspecies communication modes among human gut microbiota. Metatranscriptomic analysis showed that a number of AI-2 synthase- and receptor-encoding genes can be expressed in the human gut in healthy and/or unhealthy states. The communication network analysis suggests that AI-2-mediated interactions widely occur among members of *Firmicutes*, *Proteobacteria*, *Actinobacteriota*, *Campylobacterota*, and *Spirochaetota*. Overall, this study deepens understanding of QS-mediated communication network among human gut microbiota, and provides guidance for engineering gut microbiota and constructing new synthetic microbial consortia based on complex microbial interactions.

## Introduction

The human intestinal tract harbors the most intricate and densely populated microbial community in the body. Evidence from epidemiological, physiological, and omics studies strongly supports the notion that the indigenous gut microbiome plays a pivotal role in human health and disease [1–3]. Within this complex microbial community, microorganisms employ various strategies to interact with each other for competition, communication, and genetic exchange [4–7]. Quorum sensing (QS) is a population-level communication mechanism and diverse QS signals are employed by microbes for intraspecies and interspecies communications [8]. However, QS-based interactions remain far from fully understood in human gut microbiota.

Whereas the majority of the QS languages such as acyl-homoserine lactones (AHLs), autoinducing peptides (AIPs), and diffusible signal factors (DSFs) are species-specific, only autoinducer-2 (AI-2) and indole are reported to be able to mediate interspecies communications among bacterial species [9–12]. Indole, as a derivative of bacterial metabolism of tryptophan, remains a subject of considerable debate regarding its role as an inter-bacterial signal, mainly owing to the insufficient elucidation of its signaling receptor and precise role in coordinating bacterial population behaviors [13, 14]. Nevertheless, AI-2 stands out as a well-established QS signal that is produced by a diverse range of Gram-negative and Gram-positive bacteria and can mediate both intraspecies and interspecies communications [15]. AI-2 was shown to be able to shape the microbiota composition under conditions of dysbiosis and influence the abundance of the major phyla of the gut microbiota [16]. However, AI-2-mediated interspecies interactions among gut microbes remain largely unknown.

The AI-2 precursor 4,5-dihydroxy-2,3-pentanedione (DPD) is synthetized by S-ribosylhomocysteine lyase LuxS from S-ribosylhomocysteine, and then DPD cyclizes spontaneously into different isomers [17, 18]. Among these DPD derivatives, two active AI-2 forms engaged by the corresponding bacterial receptors have been identified, including the borated DPD derivative S-THMF-borate recognized by LuxP and the nonborated DPD derivative R-THMF recognized by LsrB [17, 18]. As two classes of periplasmic AI-2 receptors, LuxP is exclusively found in *Vibrio* spp. and LsrB is present in some enteric bacteria and members of the *Rhizobiaceae*, *Bacillaceae*, and *Clostridiaceae* families [19–21]. Upon binding to AI-2, LuxP converts the activity of the sensor histidine kinase (HK) LuxQ from kinase to phosphatase, resulting in dephosphorylation of LuxO and changing density-dependent phenotypes such as bioluminescence and biofilm formation [22]. In general, AI-2 binds to LsrB to drive Lsr activation and AI-2 internalization, and LsrB bound to AI-2 is also reported to interact with the periplasmic domain of the chemoreceptor Tsr to drive chemotactic responses in *Escherichia coli* [23–25].

Most of the bacteria that respond to AI-2 do not code for the LuxP- or LsrB-type AI-2 receptors [25–27], which has greatly hampered our understanding of the role of AI-2 in intraspecies and interspecies communications over the past two decades. Recently, our studies identified two types of transmembrane AI-2 receptors with periplasmic ligand-binding domains (LBDs), including the dCache_1 domain-containing receptors widely distributed in prokaryotes and the GAPES1 domain-containing receptors found in members of the order *Enterobacterales* [26, 27]. In particular, dCache_1-containing AI-2 receptors are found in members of more than 10 bacterial phyla and one archaeal phylum, and are comprised of all major types of signal transduction proteins in prokaryotes, including methyl-accepting chemotaxis proteins (MCPs), HKs, c-di-GMP synthases and**/**or c-di-GMP-specific phosphodiesterases (CSPs), serine phosphatases (SPs), serine/threonine kinases (STKs), and adenylate- or guanylate cyclases (ACs/GCs) [27]. The GAPES1-containing receptors are all c-di-GMP synthases homologous to YeaJ in *Salmonella* Typhimurium [26]. The discovery of these two types of widely distributed AI-2 receptors makes it possible to analyze the AI-2-mediated interspecies communications in ecosystems such as the human gut.

Here, we analyzed 3329 high-quality species-level human gut prokaryotic genomes clustered from 289232 prokaryotic genomes in the Unified Human Gastrointestinal Genome (UHGG) collection within the MGnify database and identified seven types of QS signal synthase-encoding genes in 2353 species-level prokaryotic genomes. AI-2 is the most prevalent QS signal in human gut microbiota and more than 60% prokaryotic species are found to possess the *luxS* gene. We also analyzed the presence of genes encoding the four types of AI-2 receptors and established a communication network mediated by AI-2 among members of the human gut microbiota. This study expands our understanding of AI-2-mediated interactions in human gut microbiota, providing clues for future applications such as manipulations of synthetic microbiota and potential therapies treating gut diseases.

## Materials and methods

### Bacterial strains, plasmids, and primers

Bacterial strains and plasmids used in this study are listed in Table S1. All primers used in this study were designed using Primer premier 5.0 and their sequences are listed in Table S2.

### High-quality species-level prokaryotic genomes clustered from the UHGG catalog

In this study, we used the UHGG v2 (https://ftp.ebi.ac.uk/pub/databases/metagenomics/mgnify_genomes/human-gut/v2.0/), a comprehensive reference collection of human gut microbiomes comprising 289232 prokaryotic genomes, recognized as the most extensive sequencing resource currently available for human gut microbiota. 4744 species representatives (Table S3) were obtained from the total set of 289232 prokaryotic genomes based on the species clustering analysis (dereplicated at 95% average nucleotide identity of genome sequences) as previously described [28]. The best quality genome from each species cluster was selected as the representative based on genome completeness, minimal contamination, and assembly N50 [28]. With a threshold of over 90% for completeness and <5% for contamination, we used the CheckM (v1.2.0) software [29] to select 3329 species-level genomes from the original dataset for further research.

### Distribution of 13 QS signal synthases in the human gut microbiota

We employed Prokka (v1.14.6) software [30] with default parameters to reannotate the gut microbial genomes. By delving into the annotation results generated by Prokka, the distribution characteristics of the synthases for 13 QS signals, including CqsA for cholera autoinducer-1 (CAI-1), LasI, TraI, CviI, LuxI, LuxM, EsaI, PhzI, YenI, AinS, AhyI, BafI, CarI, CepI, CinI, RaiI, HalI, NmuI, PpuI, RhiI, SinI, VanI, VanM, VfqI, YpsI, YtbI, AfeI, BjaI, ExpI, RhlI, RpaI, SmaI, SpsI, YspI, AsaI, AurI, BpsI, BraI, EcbI, EdwI, GinI, HanI, MrlI1, MrlI2, MrtI, SolI, SplI, SprI, SwrI, TofI, PagI, PcoI, SpnI, CerI, AvsI, CroI, EagI, AhlI, PsyI, BviI, CciI, AbaI, CmrI, MsaI, MlaI, EanI, YruI, AlpI, AqsI, PlaI, AcuI, NwiI, AnoI, and FilI for AHLs, LuxS or S-ribosylhomocysteine lyase for AI-2, PqsABCD/PhnAB for 4-hydroxy-2-alkylquinolines (HAQs), TnaA or tryptophanase for indole, AgrB for AIPs, Tdh or threonine dehydrogenase for 3,5-dimethylpyrazin-2-ol (DPO), AmbBCDE for 2-(2-hydroxyphenyl)-thiazole-4-carbaldehyde, PhcB for 3-hydroxypalmitic acid methyl ester/methyl-3-hydroxymyristate, LqsA for 3-hydroxypentadecan-4-one, PpyS for photopyrones, RpfF for DSFs, and DarABC for dialkylresorcinols, were investigated by using semantic approaches.

### Construction of the phylogenetic tree of species carrying QS signal synthases in the human gut microbiota

To delve deeper into the gut microbes containing QS signaling molecules, we first performed detailed genome annotations of these microbes using the Prokka software (v1.14.6) [30]. The selection of a representative species at the genus level prioritizes those that have been isolated and sequenced. For those species that have not yet undergone isolation and sequencing, the one with the highest genetic integrity within the genus is chosen as its representative. Subsequently, we inputted the gbk format files generated by Prokka into the panX [31], and constructed the phylogenetic tree of these gut microbes containing QS signal molecules using the approximate maximum likelihood method [32].

### Methods of prospecting for AI-2 receptors

Based on the annotated genomes, we analyzed the distribution of the four types of AI-2 receptors. LuxP- and LsrB-type receptors were found by searching the keywords “Autoinducer 2-binding periplasmic protein LuxP” and “the Autoinducer 2-binding protein LsrB”, respectively. The functional *lsrB* gene was further confirmed by the presence of the *lsr* operon (*lsrACDB*) and its regulator-encoding genes (*lsrK* and *lsrR*) [33]. We used the hmmsearch software [34] and the hmm definition files to preliminarily screen proteins containing the dCache_1 (PF02743) and GAPES1 (PF17155) structural domains. The dCache_1 and GAPES1 domains were further subjected to multiple sequence alignments using Muscle software (v5.1) [35]. The dCache_1-type receptors were selected based on the conservation of five residues corresponding to R126, W128, Y144, D146, and D173 in PctA of *Pseudomonas aeruginosa*, and GAPES1-type receptors were selected based on two conserved residues corresponding to Y210 and D239 in YeaJ of *S.* Typhimurium.

### Visualization of five conserved amino acid residues in 5919 dCache_1 domains within 5906 transmembrane proteins

The amino acid sequences of 5919 dCache_1 domains within 5906 transmembrane proteins from 3329 human gut prokaryotic genomes were aligned by Muscle software (v5.1) [35]. The amino acid numbering was determined based on the sequence of PctA in *P. aeruginosa*. After removing aligned columns not represented in the dCache_1 domain of PctA, the aligned sequences were visualized with WebLogo 3 [36], and the five conserved residues corresponding to R126, W128, Y144, D146, and D173 of *P. aeruginosa* PctA in those protein sequences were denoted.

### Domain annotations and functional prediction for dCache_1-type AI-2 receptors

In the previous analysis, we identified the dCache_1-type receptors within human gut microbiota. To delve deeper into the function of these proteins, we used the hmmscan software (v3.3.2) [37] to delineate structural domain and analyze potential functions of these proteins, which are classified as MCPs, HKs, CSPs, SPs, ACs/GCs, and STKs.

### In vitro AI-2 binding assays

Derivatives of pET-28a containing DNA fragments that encode the dCache_1 and GAPES1 domains were transformed into *E. coli* strain BL21 (DE3) or its mutant lacking *luxS*. The purified proteins were concentrated and denatured, and then the resulting supernatants were subjected to the *Vibrio harveyi* MM32 bioluminescence assay. Detailed method descriptions are provided in the Supplementary information.

### Isothermal titration calorimetry (ITC)

ITC experiments were carried out at 20°C using the Nano ITC Standard Volume isothermal calorimeter (TA Instruments, New Castle, DE). Seven dCache_1 domains and two GAPES1 domains were dialyzed against a Tris buffer (25 mM Tris, 300 mM NaCl, pH 8.0) and diluted to 10 μM, and DPD/AI-2 (Omm Scientific) was diluted with the same buffer to 200 μM. After being degassed, the proteins and DPD were loaded into the sample cell and syringe, respectively. Experiments were performed at 20°C with 25 injections, and the stirring speed was 200 rpm. Three independent replicates were performed for each protein. In the control experiment, the DPD/AI-2 solution (200 μM) was titrated into the sample buffer.

### Preparation of full-length transmembrane proteins with dCache_1 and GAPES1 domains

The DNA fragments encoding full-length transmembrane proteins were all cloned into a modified pET-21a vector containing N-terminal His_6_ and MBP tags, and then the constructed vectors were transformed into the Δ*luxS* mutant of *E. coli* BL21 (DE3). After IPTG induction, bacterial cells were harvested and lysed, followed by collection of the supernatants via centrifugation. The membrane fractions in the supernatants were precipitated by ultracentrifugation, followed by further purification using Ni^2+^-NTA affinity chromatography. Detailed method descriptions are provided in the Supplementary information.

### In vitro c-di-GMP synthase and phosphodiesterase activity assays

The activities of five full-length proteins in c-di-GMP synthesis or degradation were determined as described previously [26, 27]. After incubation at 30°C for 30 and 60 min in a 200-μl reaction system containing 50 mMTris–HCl (pH 7.5), 5 mM MgCl2, 70 μg of full-length protein, 100 μM substrate (GTP or c-di-GMP), and 0, 100, or 200 μM DPD/AI-2, the reaction was terminated by boiling, and the reaction products were quantified by HPLC analysis. Detailed method descriptions are provided in the Supplementary information.

### In vitro kinase assay

In vitro kinase reaction was carried out as described previously [38]. 0.5 μM full-length protein and 250 μM ATP-γ-S were incubated at 30°C for 30 min in the presence of 0, 2, or 10 μM DPD/AI-2, and then the thiophospholyation site was alkylated with 2.5 mM p-nitrobenzyl mesylate for 1 h at 30°C. The phosphorylated protein was separated by SDS-PAGE, and then detected by immunoblotting using an anti-thiophosphate ester antibody. Detailed method descriptions are provided in the Supplementary information.

### Phylogenetic trees of AI-2 synthase and receptors in the human gut microbiota

The MAFFT software [39] was used for multiple sequence alignment of the amino acid sequences of LuxS, LsrB-type AI-2 receptor, the dCache_1 domains of dCache_1-type AI-2 receptors and the full amino acid sequences of GAPES1-type receptors. Then FastTree software (v2.1.11) [32] was used to construct phylogenetic tree based on the alignment results. PctA-LBD of *P. aeruginosa* and YeaJ of *S.* Typhimurium were used as the reference sequences for the dCache_1 domains and the GAPES1-type AI-2 receptors in the phylogenetic trees, respectively. ProkkA software (v1.14.6) [30] was used to annotate all species-level genomes of the human gut microbiota that possess AI-2 receptors, and the resulting gbk files were input into panX [31] to construct a phylogenetic tree of all gut microbes containing AI-2 receptors through the maximum likelihood method. Upon completion of the phylogenetic tree construction, we employed the iTOL (v5) tool [40] for visualization analysis.

### Phyletic distribution of AI-2 synthase and receptors within the human gut microbiota

A sunburst diagram was generated to illustrate the phylogenetic distribution of species containing AI-2 synthase and receptors. The catalogue of these species was taxonomically labelled by GTDB taxonomic classification [41]. The package from ape R was used to process 2105 species that possess either the AI-2 synthase LuxS or AI-2 receptors. Following this, the iTOL (v5) tool [40] was used for visualization of the sunburst diagram.

### Human gut metatranscriptome analysis

The healthy human gut metatranscriptome data were retrieved from the NCBI SRA database, and the unhealthy human gut transcriptomic data were sourced from the publicly accessible Inflammatory Bowel Disease Multi-omics Database. After quality filtering and trimming, the data were aligned to the 3329 genomes of human gut microbiota, and the resulting SAM files were converted to BAM format. Gene counts were then carried out using FeatureCounts (v2.0.0) [42], and TPM values were computed for genes encoding AI-2 synthase and receptors. Detailed method descriptions are provided in the Supplementary information.

### Analysis of AI-2-mediated communication network in the human gut

Initially, we integrated 2105 species that possess either AI-2 synthase LuxS or AI-2 receptors, and constructed an undirected bipartite network to illustrate AI-2-mediated interspecies communications within the gut microbiome using EVenn [43]. Then, we used Cytoscape [44] to construct a communication network among 233 bacterial species possessing both LuxS and AI-2 receptors. This network includes microbes, signaling molecules, receptors, and potentially regulated downstream functions.

### Statistical analysis

GraphPad Prism 8.0 was used to perform statistical analyses. All experiments were repeated at least three times and analyzed using the two-tailed unpaired Student’s *t*-test. Data are presented as mean ± SD. ^*^P < 0.05; ^**^P < 0.01; ^***^P < 0.001.

## Results

### AI-2 is the most prevalent QS signal in the human gut microbiota

**Figure 1:**
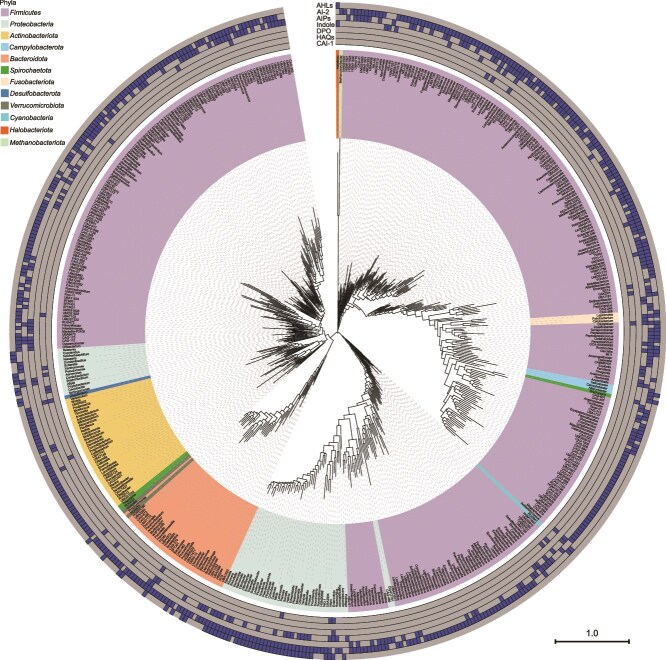
Hierarchical clustering of seven QS languages found in 543 human gut prokaryotic genera. 2353 prokaryotic species carrying genes encoding QS signal synthases are distributed in 10 bacterial phyla and two archaeal phyla and one representative species from each genus was selected for phylogenetic analysis. Bars in the outer seven layers represent the distribution of the QS languages AI-2, AIPs, AHLs, indole, HAQs, CAI-1, and DPO in the corresponding genomes, with their existence depicted in blue and absence in gray. This phylogenetic tree was constructed with the PanX software based on the genomes of 543 representative species. The scale bar represents 1.0 amino acid substitutions per site.

To investigate QS signal synthases encoded by human gut microbes, 4744 species-level human gut prokaryotic genomes were obtained from the UHGG collection comprising 289232 human gut prokaryotic genomes (Table S3). Then, 3306 bacterial genomes and 23 archaeal genomes that are >90% complete and <5% contaminated according to CheckM (Table S4) were used for search of synthase-encoding genes for 13 types of known QS signals (Table S5). 2353 species, including 2351 bacterial species and two archaeal species, were found to possess synthase-encoding genes for seven types of QS signals (Fig. S1A and Table S5). Specifically, 2038 bacterial species and one archaeal species harbored genes encoding the AI-2 synthase LuxS; 556 bacterial species carried *agrB* encoding AIP synthases; 164 bacterial species and one archaeal species possessed genes encoding AHL synthases; 215 bacterial species and one archaeal species had *tnaA* encoding for tryptophanase involved in indole production; and 18, 7, and four bacterial species contained genes encoding synthases for HAQs, CAI-1, and DPO, respectively (Fig. S1A and Table S6). Out of the 2353 prokaryotic species, 1776 species exclusively carried a single type of QS signal synthase-encoding genes, whereas 577 species harbored 2–4 types of QS signal synthase-encoding genes (Fig. 1 and Table S6).

Prokaryotic species carrying AI-2 synthase-encoding gene *luxS* exhibited widespread distribution, encompassing nine bacterial phyla and one archaeal phylum (Fig. S1B and Table S6). A total of 2067 *luxS* genes were identified across 2038 bacterial species (2066 genes) and one archaeal species (1 gene). The bacterial species carrying *luxS* were mainly distributed in *Firmicutes* (1030 species), *Actinobacteriota* (523 species), *Bacteroidota* (263 species), and *Proteobacteria* (156 species) (Figs. S1C and S2, and Table S6). Whereas the majority of bacterial species possessed single *luxS* gene, 28 bacterial species harbored dual *luxS* genes (Table S6). Additionally, the *Lachnospiraceae*, *Coriobacteriaceae*, *Bacteroidaceae*, and *Enterobacteriaceae* families were dominant within their respective phyla (Fig. S1C). Together, our analysis showed that the *luxS* gene is present across a wide range of human gut microbiota, and thus suggests that AI-2-mediated microbial communications occur widely in the human gut.

### Occurrence of genes encoding the AI-2 receptors LuxP and LsrB in the human gut microbiota

**Figure 2:**
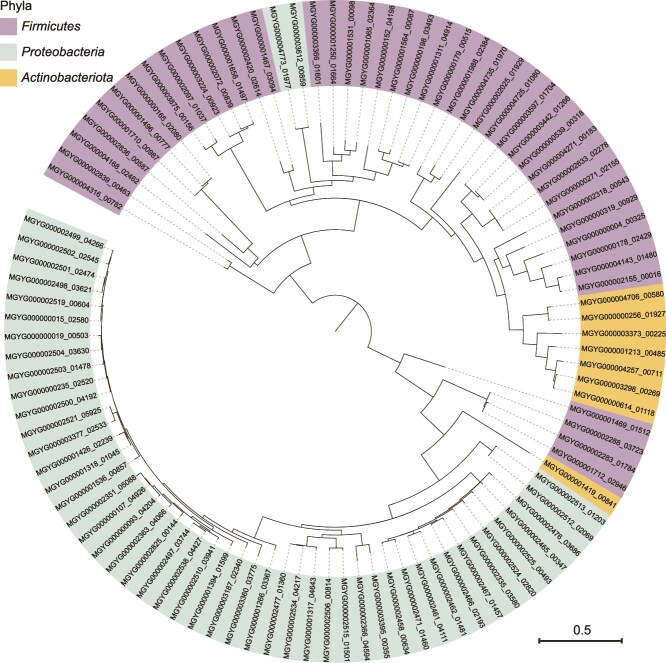
Phylogenetic analysis of LsrB-type AI-2 receptors in the human gut microbiota. This phylogenetic tree was constructed based on the amino acid sequences of 101 predicted LsrB proteins using FastTree software (v2.1.11). The scale bar represents 0.5 amino acid substitutions per site.

The QS process depends on not only the signaling molecules but also their cognate receptors [45]. First, we analyzed the distribution of genes encoding the two well-known AI-2 receptors LuxP and LsrB within the 3329 human gut prokaryotic genomes. Consistent with previous findings that LuxP is present only in *Vibrio* spp. [21, 46], the gene encoding this AI-2 receptor was only found in six *Vibrio* species (Table S7). In contrast to LuxP, the LsrB-type AI-2 receptor exhibited a relatively broad distribution range, with the *lsr* operon encoding the ABC transporter LsrACDB together with the two genes encoding its regulators LsrK and LsrR identified in 101 bacterial species. These bacterial species were thus predicted to possess a single fully functional AI-2 import system [33]. The functional LsrB-type receptors were found in *Proteobacteria* (50 species), *Firmicutes* (43 species), and *Actinobacteriota* (8 species) (Fig. 2). In addition, we found that more than half of the species harboring the functional *lsrB* gene within *Firmicutes* belonged to the *Lachnospiraceae* family*,* the species harboring functional *lsrB* within *Proteobacteria* were predominantly distributed in the *Enterobacteriaceae* family, and the species harboring functional *lsrB* within *Actinobacteriota* were mainly members of the *Atopobiaceae* family (Fig. S3). In contrast to the wide distribution of the AI-2 synthase-encoding gene in the human gut bacterial species (2038 species), genes encoding the two classical AI-2 receptors are found in a small number of bacterial species (107 species).

### Distribution of genes encoding the AI-2 receptors with dCache_1 and GAPES1 sensory domains in the human gut microbiota

**Figure 3:**
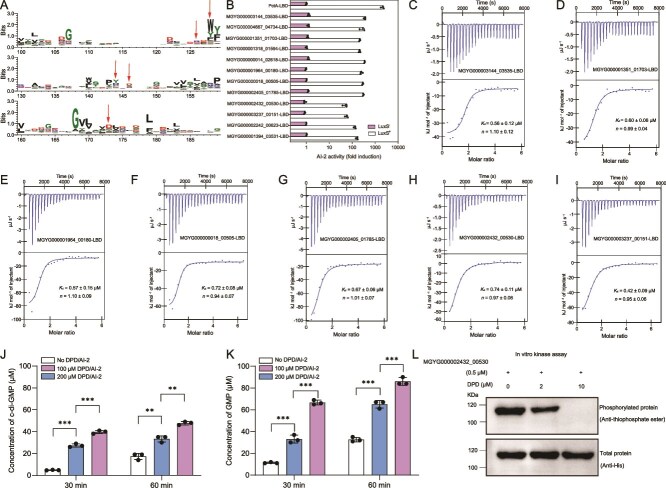
Widespread occurrence of the dCache_1-type AI-2 receptors in human gut microbiota. (A) Multiple sequence alignment and protein sequence logo of the 5919 dCache_1 domains within 5906 transmembrane proteins in 3329 human gut microbes. The five red arrows above the WebLogo denote five conserved sites corresponding to R126, W128, Y144, D146, and D173 of PctA. The sequence logos were created by WebLogo 3. (B) 12 dCache_1 domains are capable of retaining AI-2. Bioluminescence in *V. harveyi* MM32 was induced by addition of ligands released from purified proteins expressed in *E. Coli* strains with (white bars) or without the *luxS* gene (purple bars). PctA-LBD was used as a positive control. AI-2 activity is shown as fold induction relative to a buffer control. (C–I) AI-2 binds to seven dCache_1 domains with high affinity. The data are one representative of three independent experiments with similar results, with *K*_d_ and complex stoichiometry (*n*) presented as mean ± SD. (J) AI-2 induces the activity of MGYG000003144_03535 in c-di-GMP synthesis. MGYG000003144_03535 were incubated with GTP in the absence and presence of DPD/AI-2 (0, 100, and 200 μM) at 30°C before HPLC analysis. (K) AI-2 enhances the activity of MGYG000000018_00505 in c-di-GMP degradation. MGYG000000018_00505 were incubated with c-di-GMP in the absence and presence of DPD/AI-2 (0, 100, and 200 μM) at 30°C before HPLC analysis. (L) AI-2 inhibits the autophosphorylation activity of MGYG000002432_00530. MGYG000002432_00530 was incubated with ATP-γ-S in the absence and presence of DPD AI-2 (0, 2, and 10 μM) before autophosphorylation detection with anti-thiophosphate ester antibody (top). The amount of the protein was also determined by western blot with the anti-his antibody (bottom). The blots shown are one representative of three independent experiments with similar results. (B, J, K) Data are presented as mean ± SD of three independent experiments. (J, K) Statistical significance was evaluated using the Student's *t*-test. ^**^P < 0.01; ^***^P < 0.001.

**Figure 4:**
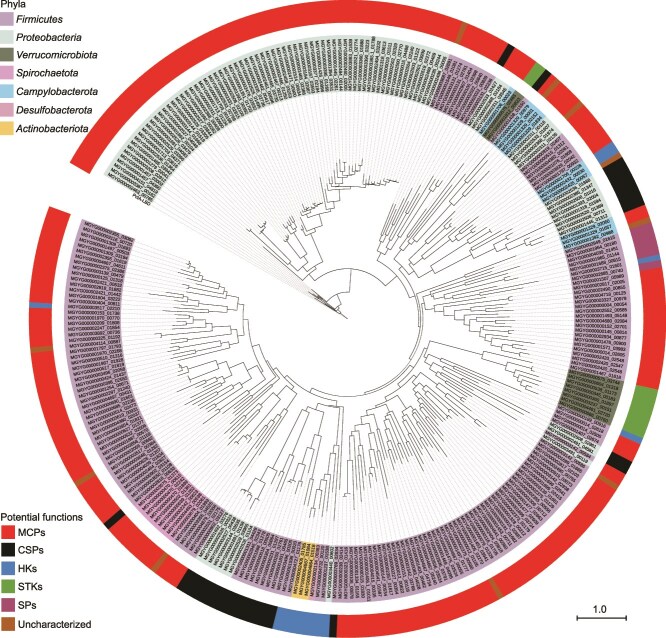
Phylogenetic analysis of 300 dCache_1 domains and the functional types of the 300 dCache_1-containing AI-2 receptors in 221 human gut bacterial species. This phylogenetic tree was constructed based on the amino acid sequences of 300 dCache_1 domains with the five conserved residues using FastTree software (v2.1.11). The outermost circle represents the functional type corresponding to each dCache_1-type AI-2 receptor. Among them, red indicates MCPs, black indicates CSPs, blue indicates HKs, green indicates STKs, purple indicates SPs, and brown indicates uncharacterized function. The scale bar represents 1.0 amino acid substitutions per site.

dCache_1 is the predominant extracellular LBD in prokaryotes and is found in all major types of transmembrane receptors, including MCPs, HKs, CSPs, SPs, STKs, and ACs/GCs [47]. In a previous study, we have identified a large group of dCache_1-containing AI-2 receptors covering each type of transmembrane signal transduction proteins in both bacteria and archaea [27]. Although the mechanisms underlying AI-2 binding by the dCache_1-type LBDs remain not yet fully understood, our previous studies have shown that dCache_1 domains that have five conserved residues corresponding to R126, W128, Y144, D146, and D173 of PctA are able to bind AI-2 [27, 48]. Domain annotations of protein sequences from the 3329 human gut prokaryotic genomes identified 5919 dCache_1 domains within 5906 transmembrane proteins (Table S8). Amino acid sequence alignment showed that 300 dCache_1 domains within 300 proteins from 221 bacterial species contained all the five conserved residues (Fig. 3A and Table S9). Among these, 288 dCache_1 domains were predicted to function as extracellular sensory modules of transmembrane receptors including MCPs (228), CSPs (30), HKs (14), STKs (10), and SPs (6), and 12 were from uncharacterized proteins without any predicted functional domains or motifs (Fig. S4 and Table S9). To confirm whether AI-2 binding is a common feature of these dCache_1 domains, 12 of them were randomly selected and subjected to the *V. harveyi* MM32 reporter assay for AI-2 binding analysis. As expected, AI-2 binding activity was observed in all these selected domains (Fig. 3B). Seven of the 12 dCache_1 domains were further subjected to AI-2 binding analysis by ITC, which confirmed the high-affinity binding of AI-2 to these dCache_1 domains (Fig. 3C–I). To verify whether AI-2 can exert regulatory effects on these dCache_1-type receptors, three full-length proteins predicted to function as CSPs and HK were expressed and purified (Fig. S5). In vitro enzymatic activity assays in the presence and absence of DPD/AI-2 followed by HPLC analysis showed that DPD/AI-2 was able to stimulate the activities of MGYG000003144_03535 and MGYG000000018_00505 in c-di-GMP synthesis and degradation, respectively (Fig. 3J and K and Fig. S6). Furthermore, DPD/AI-2 was able to inhibit the autokinase activity of MGYG000002432_00530 (Fig. 3L). These results, together with our previous findings [27], further support that the 300 dCache_1-containing proteins with the five conserved residues function as AI-2 receptors. Whereas genes encoding the largest group of dCache_1-containing AI-2 receptors MCPs were predominantly present in *Firmicutes* and *Proteobacteria*, genes encoding AI-2-sensing CSPs were found only in these two phyla (Fig. 4 and Table S9). Moreover, genes encoding AI-2-sensing HKs were distributed across *Firmicutes*, *Actinobacteriota*, *Desulfobacterota*, and *Campylobacterota*, and genes encoding AI-2-sensing STKs exclusively existed in *Verrucomicrobiota*. In addition, 52 species were found to possess 2–6 genes encoding dCache_1-containing AI-2 receptors in their genomes, and some of them harbored genes encoding two different types of transmembrane receptors (Fig. 4 and Table S9). Nevertheless, our previous studies have also shown that some dCache_1 domains not possessing all five conserved residues have the ability to sense AI-2 [27, 38], so additional AI-2 receptors must exist in the remaining 5606 dCache_1-containing proteins.

**Figure 5:**
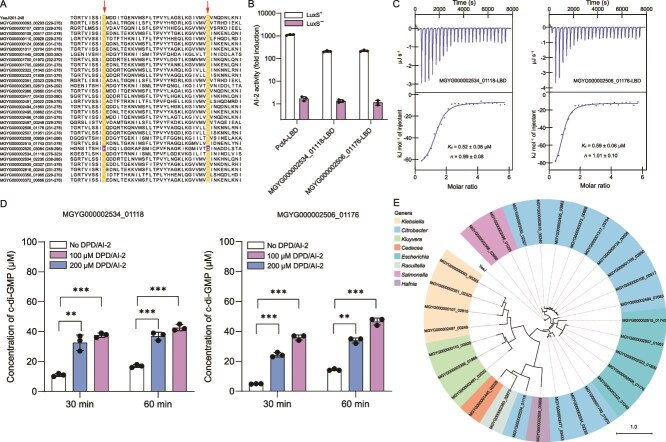
The analysis and validation of the GAPES1-type AI-2 receptors in human gut microbiota. (A) Amino acid sequence alignment of 31 GAPES1 domains and that of the YeaJ protein from *S.* Typhimurium. The conserved residues in the two positions corresponding to Y210 and D239 of YeaJ are highlighted in yellow and nonconserved residues are highlighted in purple. (B) Two GAPES1 domains are capable of retaining AI-2. Ligands released from the purified GAPES1 domains, which were expressed in *E. Coli* BL21 (DE3) with or without *luxS*, were used to induce bioluminescence in *V. *h*arveyi* MM32. PctA-LBD served as a positive control. AI-2 activity is reported as fold induction relative to light production induced by a buffer control, with values presented as mean ± SD of three independent experiments. (C) Two GAPES1 domains interact with AI-2 with high affinity. The data shown are one representative of three independent experiments with similar results. (D) AI-2 enhances the activities of MGYG000002534_01118 and MGYG000002506_01176 in c-di-GMP synthesis. The two GAPES1-containing proteins were incubated with GTP in the absence and presence of DPD/AI-2 (0, 100, and 200 μM) at 30°C before HPLC analysis. The data shown are mean ± SD of three independent experiments and the Student's *t*-test was used for statistical analyses. ^**^P < 0.01; ^***^P < 0.001. (E) Phylogenetic analysis of 30 GAPES1-type AI-2 receptors in 29 human gut bacterial species. The phylogenetic tree was constructed based on the amino acid sequences of the 30 predicted GAPES1-type AI-2 receptors using FastTree software (v2.1.11). The scale bar represents 1.0 amino acid substitutions per site.

In another study, we have identified c-di-GMP synthases harboring a GAPES1 domain with two conserved residues corresponding to Y210 and D239 of YeaJ in *S.* Typhimurium as AI-2 receptors [26]. Among the 3329 prokaryotic genomes, genes encoding 31 GAPES1 domain-containing proteins were identified across 30 bacterial species within the *Enterobacteriaceae* family (Table S10). Sequence alignment of these GAPES1 domains with that of YeaJ showed that 30 of them from 29 species contained the two conserved residues (Fig. 5A). Two GAPES1-containing proteins were randomly selected and their GAPES1 domains were found to bind AI-2 via the *V. harveyi* MM32 reporter assay and ITC analysis (Fig. 5B and C). Furthermore, the two full-length proteins were expressed and purified (Fig. S7) and then DPD/AI-2 was found to stimulate their activities in c-di-GMP synthesis (Fig. 5D and Fig. S8). These results support that the 30 GAPES1-containing proteins with the two conserved residues function as AI-2 receptors. These species harboring GAPES1-containing AI-2 receptors were members of the genera *Citrobacter*, *Escherichia*, *Klebsiella*, *Kluyvera*, *Salmonela*, *Hafnia*, *Raoultella*, and *Cedecea* (Fig. 5E and Table S10). Together, our findings suggest AI-2 will activate diverse bacterial signaling pathways upon being recognized by the extracellular dCache_1 sensors, whereas it will activate the c-di-GMP signaling pathways upon being detected by the GAPES1 sensors.

### Co-occurrence of AI-2 synthase and receptors in the human gut microbiota

Given that the four types of AI-2 receptors were found in the human gut bacterial genomes, we analyzed the comprehensive distribution patterns of all types of AI-2 receptors. 299 bacterial species were found to possess AI-2 receptors (Figs. S9A and S10, and Table S7). Bacterial species with AI-2 receptors were present in *Firmicutes*, *Proteobacteria*, *Actinobacteriota*, *Verrucomicrobiota*, *Spirochaetota*, *Campylobacterota*, and *Desulfobacterota* (Figs. S9B and S10). *Firmicutes* and *Proteobacteria* were the first and second largest phyla containing species possessing genes encoding AI-2 receptors, with the *Enterobacteriaceae* family in *Proteobacteria* containing the highest number of species harboring AI-2 receptors (77 species), followed by the *Lachnospiraceae* family in *Firmicutes* (73 species) (Table S7). Of the 299 species, 58 harbored two types of AI-2 receptors, whereas 241 had only one type of AI-2 receptors (Figs. S9C and S10).

**Figure 6:**
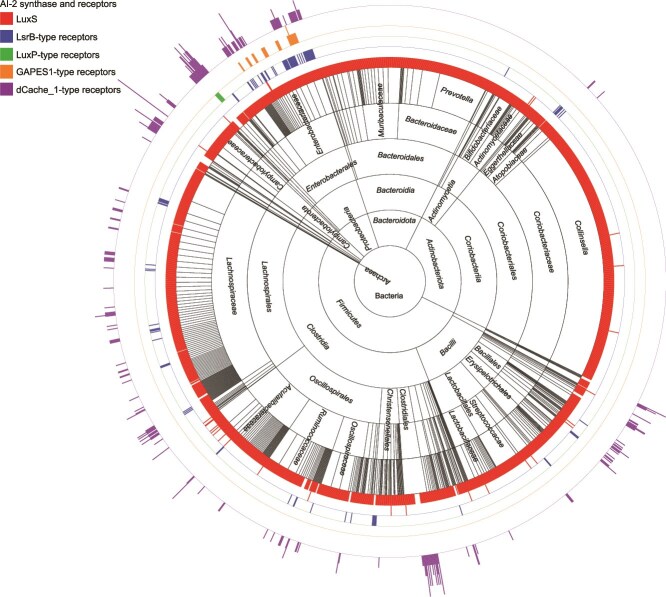
The distribution and abundance of AI-2 synthase and receptors within the 2105 human gut prokaryotic species. Blocks in the inner cycle indicate microbial taxa (kingdom, phylum, class, order, family, and genus). The red bar closest to the inner cycle indicate the distribution and number of LuxS in the corresponding prokaryotic species (2067 in 2039 species). The blue, green, orange, and purple bars in the outer layers represent the distribution and number of the LsrB-type (101 in 101 species), LuxP-type (six in six species), GAPES1-type (30 in 29 species), and dCache_1-type (300 in 221 species) AI-2 receptors, respectively, in the corresponding species. This sunburst diagram was created by ape R and visualized by iTOL.

We analyzed the co-distribution of AI-2 synthase and receptors in the human gut microbiota. 2104 bacterial species and one archaeal species were found to possess genes encoding AI-2 synthase and/or receptors (Table S11). Whereas most of these species carried *luxS*, the presence of genes encoding both LuxS and AI-2 receptors was observed in a total of 233 bacterial species, which were mainly found in *Firmicutes* and *Proteobacteria*, followed by a smaller representation in *Actinobacteriota*, *Campylobacterota*, and *Spirochaetota* (Fig. 6 and Table S11). In particular, species within *Lachnospiraceae* from *Firmicutes* and *Enterobacteriaceae* from *Proteobacteria* constituted a significant proportion at the family level (Fig. 6 and Table S11). In addition, 1805 bacterial species and one archaeal species possessed the *luxS* gene but lack AI-2 receptor-encoding genes (Table S11). These bacterial species were primarily distributed across *Firmicutes*, *Actinobacteriota*, and *Bacteroidota*. In particular, no genes encoding AI-2 receptors were identified within *Bacteroidota*, in contrast to the widespread presence of *luxS* across various species within this phylum. However, 66 bacterial species harbored genes encoding AI-2 receptors but lacked *luxS*, most of which belonged to *Clostridia* within *Firmicutes* (Table S11).

### Expression of AI-2 synthase and receptors in human gut metatranscriptome datasets

Given that particular bacterial species possess the genetically determined potential to produce and/or perceive AI-2 does not necessarily mean these species interact within the gut [48, 49], whether the abundance of AI-2 synthase and receptors would even allow meaningful interaction remains to be further explored. To investigate whether the identified *luxS* and AI-2 receptor-encoding genes within the 2105 species-level genomes are actually expressed in the human gut, we analyzed their expression within 20 healthy and 20 unhealthy human gut metatranscriptome datasets (Table S12). In the healthy datasets, 834 genomes expressed *luxS*, distributed across four bacterial phyla including *Actinobacteriota* (401 species), *Firmicutes* (314 species), *Bacteroidota* (83 species), and *Proteobacteria* (36 species) (Table S12). Relatively high *luxS* expression (TPM > 0.2) was observed in 77 species within *Bacteroidota*, *Firmicutes*, and *Actinobacteriota*, whereas all species within *Proteobacteria* showed relatively low expression of this gene (Fig. S11 and Table S12). 105 bacterial species expressed AI-2 receptor-encoding genes within the healthy datasets (Table S12). Among them, 41 species expressed *lsrB*, 63 species expressed the dCache_1-type receptor-encoding genes, and two species expressed the GAPES1-type receptor-encoding genes. However, only 11 species expressed AI-2 receptor-encoding genes at a level (TPM > 0.2) comparable with that of *luxS* (Fig. S11 and Table S12). Co-expression of the AI-2 synthase and receptors was identified in 26 species, which were predominantly distributed in the *Lachnospiraceae* family (19 species) within *Firmicutes* (Table S12). In the unhealthy datasets, the number of species co-expressing AI-2 synthase and receptors increased to 70, with a shift from species located predominantly within *Lachnospiraceae* in the healthy state to species located predominantly within *Enterobacteriaceae* in the unhealthy state (Table S12). Although the number of the species expressing AI-2 synthase and receptors increased in the unhealthy datasets compared with the healthy datasets, the number of species with relative high expression of LuxS (TPM > 0.2), LsrB- (TPM > 0.1), and dCache_1-type AI-2 receptors (TPM > 0.01) drastically decreased (Fig. S12 and Table S12). In fact, a metatranscriptome profile is only a snapshot of the transcriptional activity at a specific point in time under certain environmental conditions, and the expression levels are proportional to the abundance of bacteria within the microbial community. Nevertheless, our analysis at least shows that many species within the human gut can express AI-2 synthase and receptors to communicate with each other.

### AI-2 mediates widespread communications in the human gut microbiota

**Figure 7:**
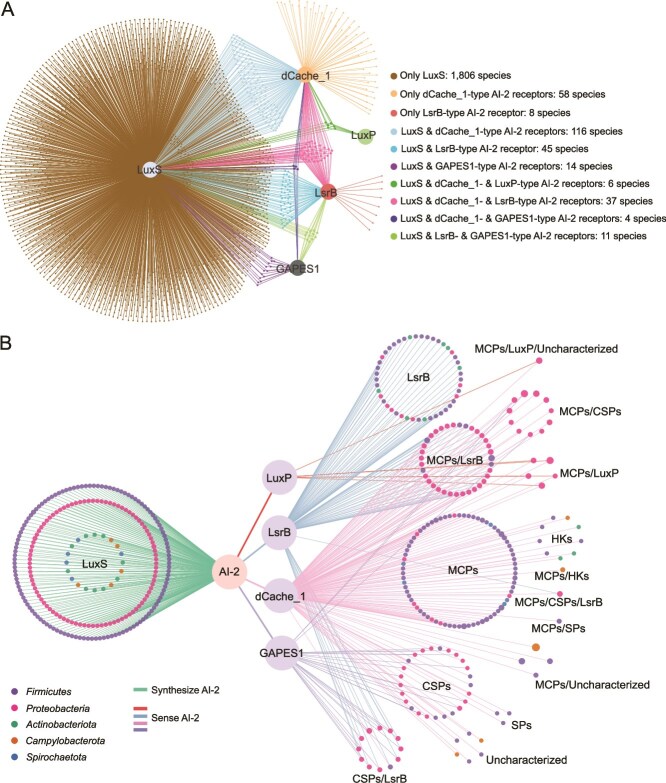
AI-2 mediates widespread communication in the human gut microbiota. (A) AI-2-mediated interspecies communications network for 2105 species carrying the AI-2 synthase and/or receptors within human gut microbiota. The nodes in this network represent the 2104 bacterial species and one archaeal species. The color of the node represents the presence of LuxS and AI-2 receptors within this species. The edges symbolize the potential communication processes (the synthesis and sensing of the AI-2 signal). (B) AI-2-mediated communication network among 233 bacterial species with both LuxS and AI-2 receptors. In this network, nodes represent species with their different colors indicating the phylum to which the species belongs, whereas the size of the nodes reflects the number of LuxS or AI-2 receptors of each functional type in the species. Green edges symbolize the synthesis of AI-2 by species, and the orange, blue, pink, and gray-purple edges indicate the perception of AI-2 by the four types of receptors to activate the functional modules (LuxP, LsrB, MCPs, CSPs, HKs, and SPs).

To elucidate AI-2-mediated communication network within the human gut, we initially constructed an undirected bipartite network, which presented AI-2-mediated potential interspecies communications among the 2104 bacterial species and one archaeal species that possess AI-2 synthase and/or receptors (Fig. 7A and Table S11). The nodes in this communication network represent the 2105 prokaryotic species, and the edges represent potential communication processes including the synthesis and sensing of the AI-2 signal (Fig. 7A). 233 human gut bacterial species can not only produce AI-2 but also sense this signal secreted by itself and other bacterial species. In contrast, 1805 bacterial species and one archaeal species can only produce AI-2, but do not harbor an AI-2 receptor to sense this signal, whereas 66 bacterial species that do not produce AI-2 can sense this signal via one or two types of receptors (Fig. 7A). Although more unknown AI-2 receptors, especially the dCache_1-type receptors, in the human gut microbiota are yet to be discovered, the communication network suggests that AI-2 can mediate extensive and complex interspecies communications among the human gut microbiota.

Considering the aforementioned 233 bacterial species possessing both LuxS and AI-2 receptors may play an important role in QS within the human gut, AI-2-mediated communication network among these species was further established based on the types and functions of AI-2 receptors (Fig. 7B and Table S13). In this network, nodes symbolize species, green edges show the generation of AI-2 by bacterial species, and orange, blue, pink, and gray-purple edges indicate the perception of AI-2 by the four types of receptors to activate the functional modules and exert regulatory effects. Except the dCache_1- (MCPs, CSPs, HKs, and SPs) and GAPES1-type (CSPs) AI-2 receptors with clear functional modules, AI-2 binds to LuxP to modulate the activity of the sensor HK LuxQ in *Vibrio* spp. [22], whereas AI-2 generally binds to LsrB to drive Lsr activation and AI-2 internalization, and also mediates chemotactic responses in *E. coli* via interaction with the chemoreceptor Tsr [23]. AI-2-mediated communications were shown to occur among species within the phyla *Firmicutes*, *Proteobacteria*, *Actinobacteriota*, *Campylobacterota*, and *Spirochaetota*. All of these species were predicted to secrete AI-2 for communication, and members of *Firmicutes* and *Proteobacteria* can sense this signal via one to three kinds of functional modules belonging to LuxP, LsrB, MCPs, CSPs, HKs, or SPs. In addition, members of *Actinobacteriota* can sense AI-2 via LsrB and HKs, those of *Campylobacterota* can sense AI-2 via MCPs and HKs, and those of *Spirochaetota* can sense AI-2 via MCPs. Moreover, 56 species within *Proteobacteria*, 11 species within *Firmicutes* and two species within *Campylobacterota* possessed two different kinds of functional modules to detect AI-2. Sensing of AI-2 by functionally distinct receptors will lead to different cellular responses. Thus, our results suggest not only widespread occurrence of AI-2-mediated interspecies communications within the human gut microbial communities, but also the diversity of interspecies communication modes among human gut microbiota.

## Discussion

As a ubiquitous communication process among microorganisms, QS endows microbial cells with the ability to communicate with each other using chemical molecules, thereby coordinating their activities in response to changes in population density and species composition [45, 50]. Understanding of QS-based complex microbial interactions in human gut microbiota will be of vital importance to engineer the gut microbiota and regulate the microbial interactions as therapeutics for treating gut diseases [15]. However, to date, the types, distribution, and abundance of QS systems present in the human gut microbiota still remain little explored. Highly redundant microbial genomes as well as insufficient microbial diversity greatly hamper understanding of the roles of individual microbial species and their interactions [28, 51]. The UHGG collection is the most comprehensive sequence resources of the human gut microbiome established thus far and comprises 289232 nonredundant prokaryotic genomes that were clustered into 4744 species representatives (https://www.ebi.ac.uk/metagenomics/genome-catalogues/human-gut-v2-0-2) [28]. In the present study, by analyzing the 3329 high-quality species-level human gut prokaryotic genomes in the UHGG collection, we show that AI-2 is the most prevalent QS signal within the human gut microbiota. Our results also reveal the wide distribution of AI-2 receptor-encoding genes and the functional diversity of AI-2 receptors, thus highlighting the complexity of AI-2-mediated interspecies communication network within the human gut microbiota.

The human gut microbiota is an “invisible organ” in the body and has been implicated in various important phenotypes related to human health and disease [1, 52, 53]. However, as the main mechanism of bacterial cell-to-cell communication, QS-based interspecies interactions remain poorly understood, largely due to the fact that most QS signals are dedicated to intraspecies communication. Although indole and AI-2 have been recognized as interspecies QS languages in the past two to three decades, both of them face serious challenges in terms of the signal properties [14, 45, 46, 54]. Whereas a number of bacteria have been shown to produce large quantities of indole, there is no clearly defined protein receptor or mode of action for this signaling molecule in bacterial cells [14, 54]. Thus, although our study shows that more than 200 human gut bacterial species harbor the *tnaA* gene responsible for indole production, it is still difficult to investigate indole-mediated interspecies communications among the human gut microbiota. In addition to the yet enigmatic signal indole, AI-2 is the only QS signal shared by both Gram-positive and Gram-negative bacteria so far, and previous studies have shown that it is more widely distributed than indole in the rumen and human gut microbiota [48, 55]. In contrast to the wide distribution of AI-2, its two well-known receptors LuxP and LsrB have only been found in a small number of bacterial species [19, 56, 57]. Due to the narrow distribution of LuxP and LsrB in bacteria, whether AI-2 can be defined as a true interspecies signal has been questioned since its discovery [45, 46, 58]. Nevertheless, our two recent studies identified dCache_1- and GAPES1-containing signal transduction proteins as AI-2 receptors, with the former widely distributed in prokaryotes and the latter found in 17 genera of the order *Enterobacterales* [26, 27], thereby providing evidence supporting the view that AI-2 can serve as an interspecies signal allowing widespread communication between bacterial genera*.* The dCache_1-type LBDs are the predominant extracellular sensor domains in prokaryotes and are found in all major families of prokaryotic transmembrane receptors [27, 47], providing a huge resource for discovering unknown AI-2 receptors. Whereas the present study has identified 300 dCache_1-type AI-2 receptors with five conserved residues from 221 human gut bacterial species, our recent studies also identified several dCache_1-type AI-2 receptors not possessing all five conserved residues from bacterial species such as *P. aeruginosa*, *Rhodopseudomonas palustris*, *Bacillus subtilis*, and *V. furnissii* [27, 38], suggesting that more AI-2 receptors remain to be discovered from the more than 5000 dCache_1-containing transmembrane proteins not possessing all five conserved residues encoded by the gut prokaryotic species. Whereas our work has shown that the dCache_1-containing AI-2 receptors are the largest type of receptors for this signal in the human gut microbiota, it is expected that the number of this type of receptors will continue to grow.

Our study shows that AI-2-mediated interspecies communications in the human gut microbiota are not only widespread but also diverse in the mode of action. Before the discovery of dCache_1-type AI-2 receptors, QS-mediated chemotaxis has only been reported for AI-2 and indole in several Gram-negative bacterial species including *E. coli* and *Helicobacter pylori* [23, 59–62]. MCPs have been shown to be the most abundant and widely distributed dCache_1-containing signal transduction proteins in prokaryotes [27, 47], and our results show that dCache_1-containing MCPs are also the largest functional group of AI-2 receptors that are distributed across four phyla including Gram-positive and Gram-negative bacteria, suggesting that AI-2-mediated chemotaxis widely occurs and might be crucial for the gut microbiota to occupy a favorable ecological niche in the gut. Given that numerous dCache_1-type AI-2 receptors are not fully explored, LsrB currently represents the second largest functional group of AI-2 receptors in the gut microbiota. Despite being found to play a role in chemotaxis, cell aggregation, and biofilm formation in *E. coli* [24, 59, 62], LsrB was shown to mediate AI-2 internalization and result in a rapid depletion of AI-2 from the extracellular medium in most cases [20, 56, 63], suggesting that this type of AI-2 receptors mainly functions to prevent excessive AI-2 level in the extracellular environment. CSPs that catalyze the synthesis and degradation of c-di-GMP constitute the third most abundant functional group among AI-2 receptors in the human gut microbes, including both dCache_1- and GAPES1-containing CSPs. As a near-ubiquitous second messenger in bacteria, c-di-GMP controls various important biological processes, including, but not limited to, biofilm formation, motility, cellular differentiation, virulence, and stress adaptation [38, 64–67]. 54 species within *Proteobacteria* and *Firmicutes* harbor AI-2-sensing CSPs, and the majority of these species are distributed in the *Enterobacteriaceae* family (Fig. 5E and Table S9), suggesting members of this family in the gut microbiota tend to sense the AI-2 signal to coordinate their growth and behavior via c-di-GMP signaling pathways. Although other functional groups of AI-2 receptors such as HKs, STKs, and SPs have only been found in a very small number of species (Fig. 4), these functional groups sensing AI-2 can be further found from those unexplored dCache_1-containing transmembrane proteins not possessing all five conserved residues. Therefore, the complex modes of communication mediated by the AI-2 signal remain to be further explored in the gut microbiota.

In summary, our study suggests that AI-2-based QS system is the main QS system that mediates interspecies interactions in the human gut. The AI-2 communication network within the human gut microbiota established in this study helps to understand the communication process mediated by AI-2 at a holistic level. The extensiveness and complexity of AI-2-mediated potential communications suggest an important role of AI-2 signaling in microbial interactions in human gut. Our work provides an important theoretical and practical reference for exploring the role of AI-2-based microbial interactions in human health and disease. In contrast to the widespread distribution of this signal in the human gut microbiota, AI-2 receptors are far from being fully explored and thus our understanding of AI-2-mediated potential interspecies interactions in the gut is still insufficient. Nevertheless, our study represents an important resource for future mechanistic investigations that may offer deeper insights into the roles of AI-2 signaling in the gut.

## Supplementary Material

Clean_revised_Supplementary_information_wraf204

digital_version_of_Figure_S2_including_all_protein_labels_wraf204

Table_S1_wraf204

Table_S2_wraf204

Table_S3_wraf204

Table_S4_wraf204

Table_S5_wraf204

Table_S6_wraf204

Table_S7_wraf204

Table_S8_wraf204

Table_S9_wraf204

Table_S10_wraf204

Table_S11_wraf204

Table_S12_wraf204

Table_S13_wraf204

## Data Availability

The prokaryotic genome assemblies used in this study were obtained from the UHGG v2.0.2 catalog available at the associated FTP site (https://ftp.ebi.ac.uk/pub/databases/metagenomics/mgnify_genomes/human-gut/v2.0.2/) of the MGnify database. The healthy human gut transcriptomic data utilized in this study were retrieved from the NCBI SRA database with accession numbers SRR6038380, SRR6038379, SRR6038382, SRR6038381, SRR6038376, SRR6038375, SRR6038378, SRR6038377, SRR6038488, SRR6038489, SRR6038486, SRR6038487, SRR6038484, SRR6038485, SRR6038482, SRR6038483, SRR6038480, SRR6038481, SRR6038196, and SRR6038210. The unhealthy human gut transcriptomic data were sourced from the publicly accessible Inflammatory Bowel Disease Multi-omics Database (https://ibdmdb.org/) with accession numbers CSM9X23N, HSMA33KE, PSM7J18I, CSM79HR8, HSM7J4QT, HSMA33J3, MSMB4LXW, PSM7J18E, PSMA2668, MSM79HA3, HSM5MD6K, MSMAPC5L, CSM67UBH, MSM9VZNX, MSM79H5Q, MSM6J2RS, CSM67UEW, CSM7KOLA, PSM7J1BB, and CSM79HHO. All data generated or analysed during this study are included in this published article and its supplementary information files.
