# Supplementary material for: Autoinducer-2-mediated communication network within human gut microbiota
Source: ISME J. 2025 Sep 11;19(1):wraf204. doi: 10.1093/ismejo/wraf204 (PMC12503165; doi:10.1093/ismejo/wraf204)
Supplement: Clean_revised_Supplementary_information_wraf204 [file clean_revised_supplementary_information_wraf204.docx]

**Supplementary Information for**

**AI-2-Mediated Communication Network within** **Human Gut** **Microbiota**

Qingying Fan^1†^, Hengxi Sun^2†^, Xueyuan Lin^1†^, Wenguang Yang^1^, Xihui Shen^1^, Lei Zhang^1^*

^1^State Key Laboratory for Crop Stress Resistance and High-Efficiency Production, Shaanxi Key Laboratory of Agricultural and Environmental Microbiology, College of Life Sciences, Northwest A&F University, Yangling, Shaanxi 712100, China.

^2^MOE Key Laboratory of Contemporary Anthropology, Department of Anthropology and Human Genetics, School of Life Sciences, Fudan University, Shanghai 200438, China

†These authors contributed equally to this work.

**This file includes:**

**Supplementary Material and Methods**

**Supplementary Figure. S1 to S12**

**Supplementary References**

**Supplementary Material and Methods**

**Human gut metatranscriptome analysis**

Healthy human gut metatranscriptome data utilized in this study were retrieved from the Sequence Read Archive (SRA) database at the National Center for Biotechnology Information (NCBI). For comparative analysis, inflammatory bowel diseases (IBD) were selected as a representative of unhealthy conditions, and the data were sourced from the publicly accessible Inflammatory Bowel Disease Multi'omics Database (https://ibdmdb.org/). In both the healthy and unhealthy groups, data selection was performed randomly, with 20 datasets chosen for each group. In the healthy group, 20 distinct samples were selected, corresponding to accession numbers SRR6038380, SRR6038379, SRR6038382, SRR6038381, SRR6038376, SRR6038375, SRR6038378, SRR6038377, SRR6038488, SRR6038489, SRR6038486, SRR6038487, SRR6038484, SRR6038485, SRR6038482, SRR6038483, SRR6038480, SRR6038481, SRR6038196, and SRR6038210 [1]. In the unhealthy group, the accession numbers corresponding to 20 different samples were CSM9X23N, HSMA33KE, PSM7J18I, CSM79HR8, HSM7J4QT, HSMA33J3, MSMB4LXW, PSM7J18E, PSMA2668, MSM79HA3, HSM5MD6K, MSMAPC5L, CSM67UBH, MSM9VZNX, MSM79H5Q, MSM6J2RS, CSM67UEW, CSM7KOLA, PSM7J1BB, and CSM79HHO [2]. All samples were sequenced with high throughput paired-end sequencing using the HiSeq 2000 System (Illumina) [1, 2]. The FastQC (version 0.11.9) and MultiQC (version 1.9) [3] toolkits were initially employed to conduct a quality assessment of the metatranscriptome data, and low-quality bases in the raw data were trimmed utilizing Trimmomatic (version 0.39) (LEADING:3 TRAILING:3 SLIDINGWINDOW:4:20 MINLEN:50) [4]. After trimming, the data were aligned to the 3,329 genomes of human gut microbiota using Bowtie2 (v2.3.5.1) [5], and the resulting SAM format alignment files were converted to BAM format using SAMtools (v1.10) [6]. Gene counts were then carried out using FeatureCounts (v2.0.0) [7]. Finally, we computed these counts to generate a table of TPM values, from which we derived the statistical data for genes encoding AI-2 synthase and receptors.

**In vitro AI-2 binding assays**

The DNA fragments encoding dCache_1 domains of 12 predicted dCache_1-type AI-2 receptors and GAPES1 domains of 2 predicted GAPES1-type AI-2 receptors were synthesized by Genewiz (Suzhou, China). Derivatives of pET-28a containing DNA fragments that encode the dCache_1 domains and GAPES1 domains were transformed into *E. coli* strain BL21(DE3) or its mutant lacking *luxS*. These resulting strains were cultivated in LB medium at 37°C until reaching an OD_600_ of 0.8, at which time the temperature was transferred to 20°C and 0.25 mM IPTG was added to induce protein expression for 7 h. The cells were then lysed by sonication and His_6_-tagged recombinant proteins were purified using Ni-nitrilotriacetic acid (Ni^2+^-NTA) His-binding resin in accordance with the protocol of the manufacturer (Novagen, Madison, WI). Then, the eluted proteins were swapped into a solution containing 50 mM NaH_2_PO_4_ (pH 8.0), 300 mM NaCl and 1 mM dithiothreitol using Sephadex-G25 agarose. Following confirmation of purity through SDS-PAGE analysis, the purified proteins were concentrated to approximately 10 mg ml^−1^ and denatured by heating at 70°C for 10 min to release any bound ligands. The denatured proteins were precipitated, and the resulting supernatants were subjected to the *Vibrio* *harveyi* MM32 bioluminescence assay. The measurement of AI-2 activity was carried out using microplate reader Victor X3 (PerkinElmer, Waltham, MA, USA), which was operated through the PerkinElmer 2030 Workstation 4.0.

**Preparation of full-length transmembrane proteins with dCache_1 and GAPES1 domains**

The DNA fragments encoding the full-length proteins MGYG000003144_03535, MGYG000000018_00505, and MGYG000002405_01785 containing the dCache_1 domain, as well as the proteins MGYG000002534_01118 and MGYG000002506_01176 containing the GAPES1 domain, were synthesized by Genewiz (Suzhou, China). The amplified DNA fragments of these proteins were all cloned into a modified pET-21a vector, which contained an N-terminal His_6_ tag followed by a maltose-binding protein (MBP) tag. Subsequently, these constructed vectors were transformed into the Δ*luxS* mutant of *E. coli* BL21(DE3). The resulting bacteria strains were grown at 37°C in LB medium, and protein expression was induced at OD_600_ of 0.8 by adding 0.5 mM IPTG, followed by incubation at 16℃ for 10 h. Bacterial cells were collected and then lysed in the lysis buffer (20 mM Tris-HCl, pH 8.0; 300 mM NaCl; 10% glycerol), followed by centrifugation at 10,000 × *g* for 1 h at 4°C. Subsequently, the supernatants were ultracentrifuged at 4℃ and 200,000 × *g* for 1 h. The membrane fractions were resuspended using a high-salt buffer (20 mM Na_3_PO4, pH 7.0; 2 M KCl; 10% glycerol; 5 mM EDTA; 5 mM DTT; 1 mM phenylmethanesulfonyl fluoride). After resuspension, they were ultracentrifuged at 350,000 g for 1 h, and this process was repeated twice. The obtained membrane fractions were finally purified by Ni^2+^-NTA affinity chromatography, and the protein purity was evaluated by SDS-PAGE. The amount of the finally obtained proteins in membrane fractions was determined by Bradford assay.

**In vitro c-di-GMP synthase activity assays**

The activities of MGYG000003144_03535, MGYG000002534_01118, and MGYG000002506_01176 in c-di-GMP synthesis were determined as described previously [8, 9]. 70 μg of the full-length protein, 100 μM GTP, and 0, 100, or 200 μM DPD/AI-2 were added to a 200-μl reaction system containing 50 mM Tris-HCl (pH 7.5) and 5 mM MgCl_2_. The reactions were conducted at 30°C. At 30 and 60 min after the reaction initiation, 50 μl aliquots were taken and heated at 100°C for 5 min. Denatured proteins were removed by centrifugation and the remaining supernatants were filtered through a 0.22 μm membrane. The supernatants were then loaded onto a reverse-phase C18 column and separated by HPLC. The detection wavelength was 254 nm. GTP (Sigma, Cat# G8877) and c-di-GMP (Sigma, Cat# SML1228) were run as standards and the c-di-GMP levels in the supernatants were determined from a standard curve established with a serially diluted c-di-GMP solution.

**In vitro c-di-GMP-specific phosphodiesterase activity assays**

The activity of MGYG000000018_00505 in c-di-GMP degradation was determined using 70 μg of protein and 100 μM c-di-GMP in the presence of 0, 100, or 200 μM DPD/AI-2 in 200 μl of reaction buffer containing 50 mM Tris HCl (pH 7.5) and 5 mM MgCl_2_. Samples were harvested after incubation for 30 and 60 min and then boiled for 5 min to stop the reaction. Denatured protein was removed by centrifugation after which the supernatant was filtered through a 0.22 μm membrane. The supernatant was loaded onto a reverse-phase C18 column and separated by HPLC [10]. GMP (Sigma, Cat# G8377) and c-di-GMP (Sigma, Cat# SML1228) were used as standards. The levels of GMP produced in the reaction were determined based on the standard curve established with known concentrations of GMP.

**In vitro kinase assay**

In vitro kinase reaction was carried out as described previously [10]. 0.5 μM full-length MGYG000002432_00530 and 250 μM ATP-γ-S (ab138911, Abcam) were incubated in the presence of 0, 2, or 10 μM DPD/AI-2 in a 20-μl reaction system containing 50 mM Tris (pH 7.4) and 10 mM MgCl_2_. After reaction for 30 min at 30℃, the thiophospholyation site on MGYG000002432_00530 was alkylated with 2.5 mM p-nitrobenzyl mesylate (ab138910, Abcam) for 1 h at 30℃, and then the reaction was stopped with 5×SDS loading buffer. The thiophosphate ester-modified protein was separated by SDS-PAGE and transferred to PVDF membranes using a wet blotting system (Bio-Rad). After blocking with QuickBlockBlocking Buffer at room temperature for 1 h, the membranes were incubated overnight at 4℃ with rabbit anti-thiophosphate ester antibody (ab92570, Abcam). After washing with TBST for six times, the membranes were incubated with goat anti-rabbit horseradish peroxidase-conjugated secondary antibody (1:5,000 dilution, Cat No. SA00001-2, Proteintech) for 1 h at room temperature. Finally, the membranes were washed six times with TBST and visualized using an ECL detection reagent. For control, the total protein amount was also determined by incubation first with 1:5,000 dilution of mouse anti-His antibody (Abways, Shanghai, China, cat# AB0002), followed by incubation with 1:10,000 dilution of goat anti-mouse horseradish peroxidase-conjugated secondary antibodies (DIYIBIO, China, cat# DY60203). The signals were also detected using the ECL system.

**Supplementary Figures (Figure S1-S12)**

**
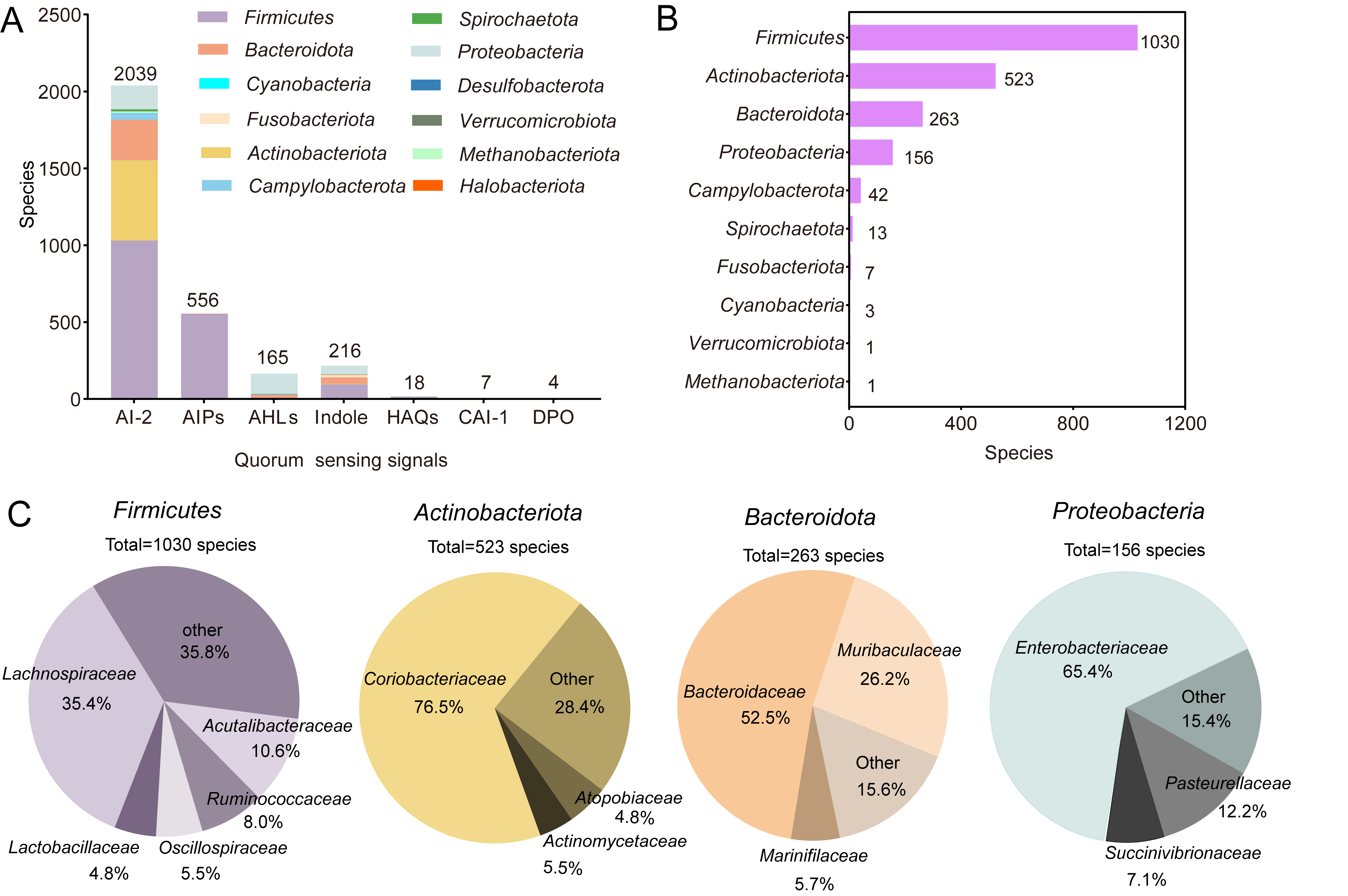
**

**Figure S1.** Distribution of seven QS signals among 2,353 prokaryotic species in the human gut microbiota. **A**. Numbers and distribution of prokaryotic species possessing AI-2, AIP, AHL, Indole, HAQs, CAI-1, and DPO signals. **B**. The phylum-level distribution of *luxS*-possessing species in the human gut microbiota. **C.** Dominant families of *luxS*-possessing species within *Firmicutes*, *Actinobacteriota*, *Bacteroidota*, and *Proteobacteria*.


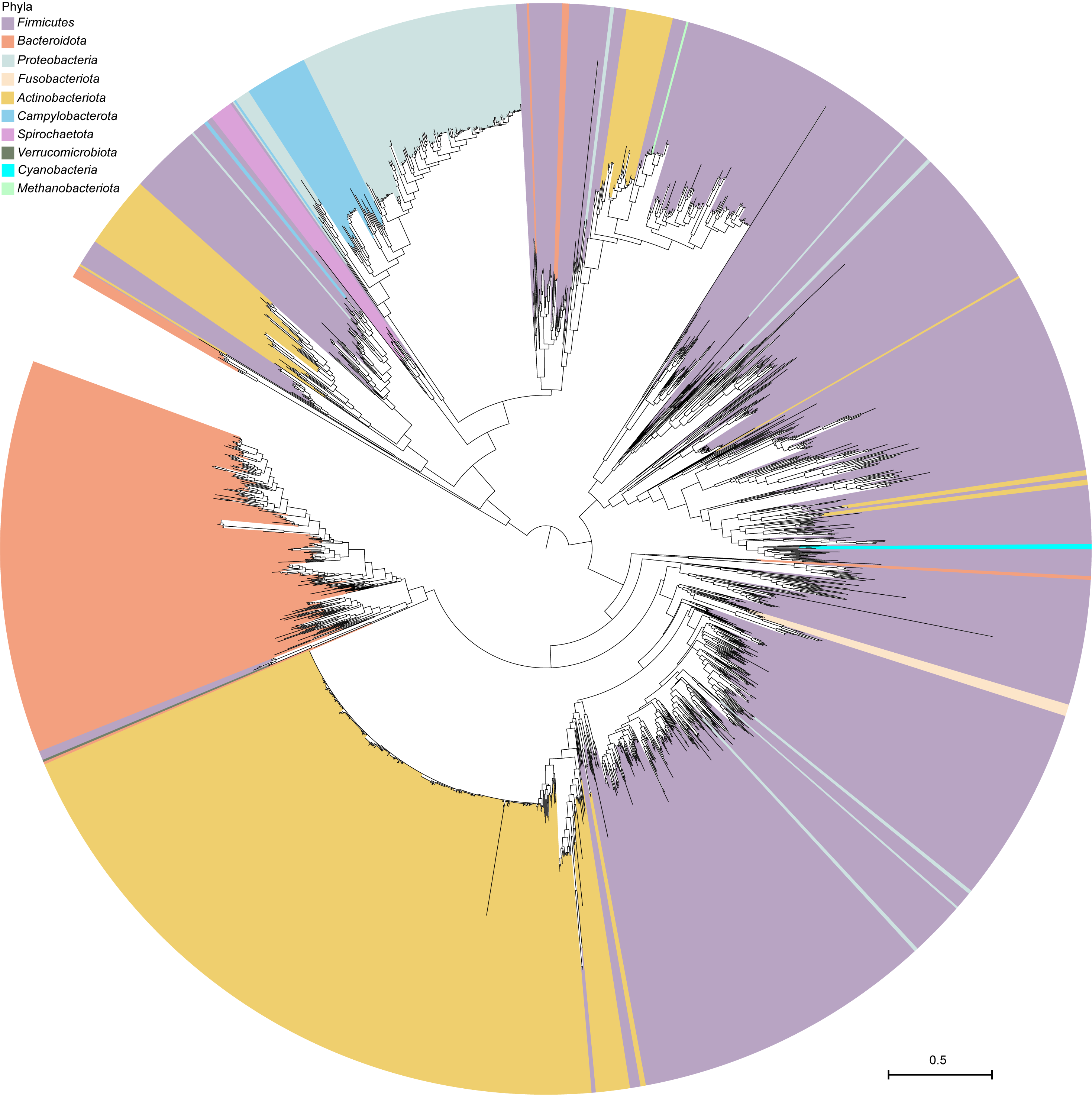


**Figure S2.** Phylogenetic analysis of the AI-2 synthase LuxS in the human gut microbiota. 2,067 *luxS* genes were found from 2,039 human gut prokaryotic species that are distributed in 9 bacterial phyla and 1 archaeal phylum. This phylogenetic tree was constructed based on the amino acid sequences of the predicted LuxS proteins using FastTree software (v2.1.11). All protein labels of LuxS have been removed from the tree, and a digital version of the same tree including all protein labels is available in electronic supplementary material. The scale bar represents 0.5 amino acid substitutions per site.

**
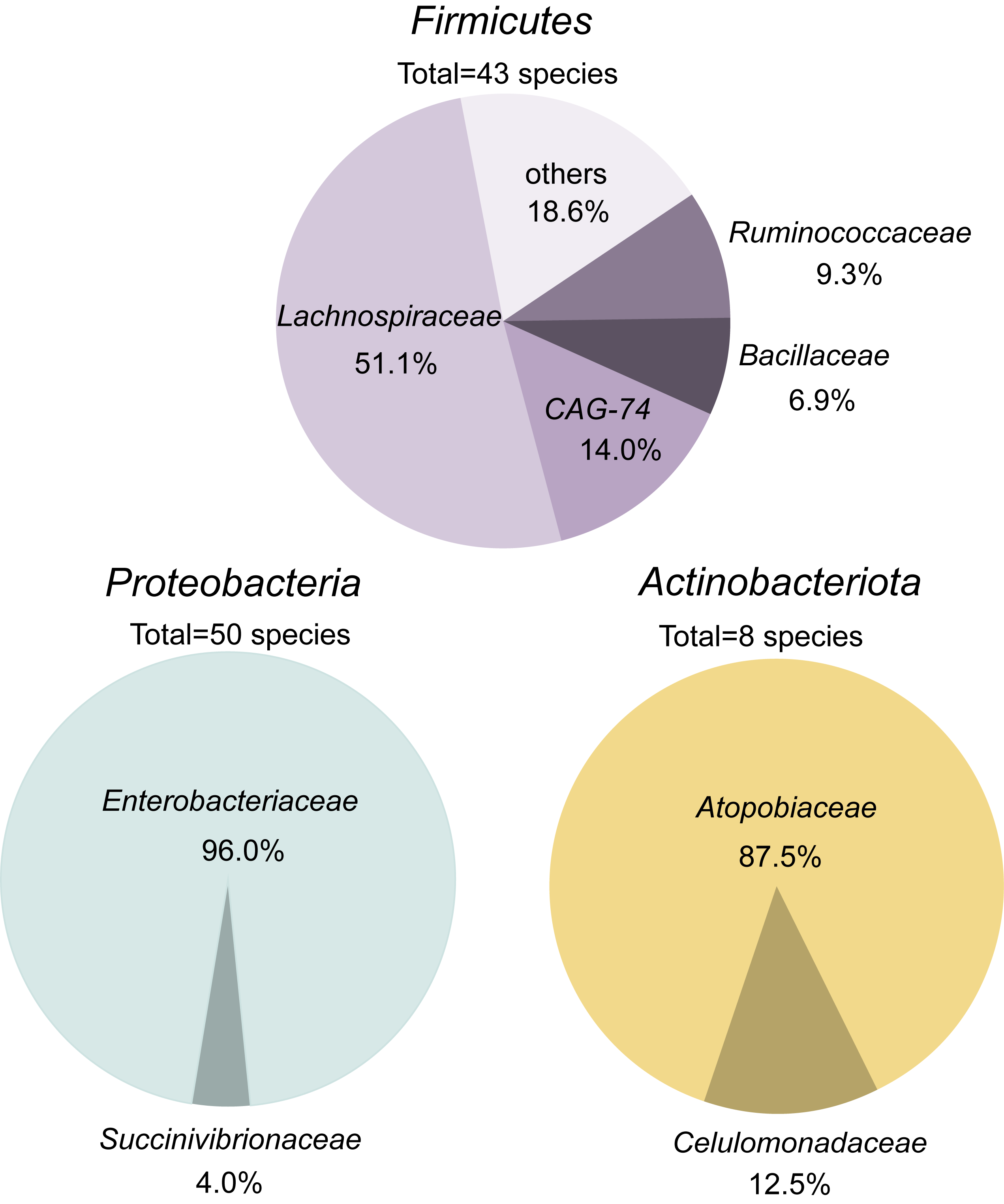
**

**Figure S3.** The family-level distribution of bacterial species possessing the LsrB-type AI-2 receptor within human gut microbiota.


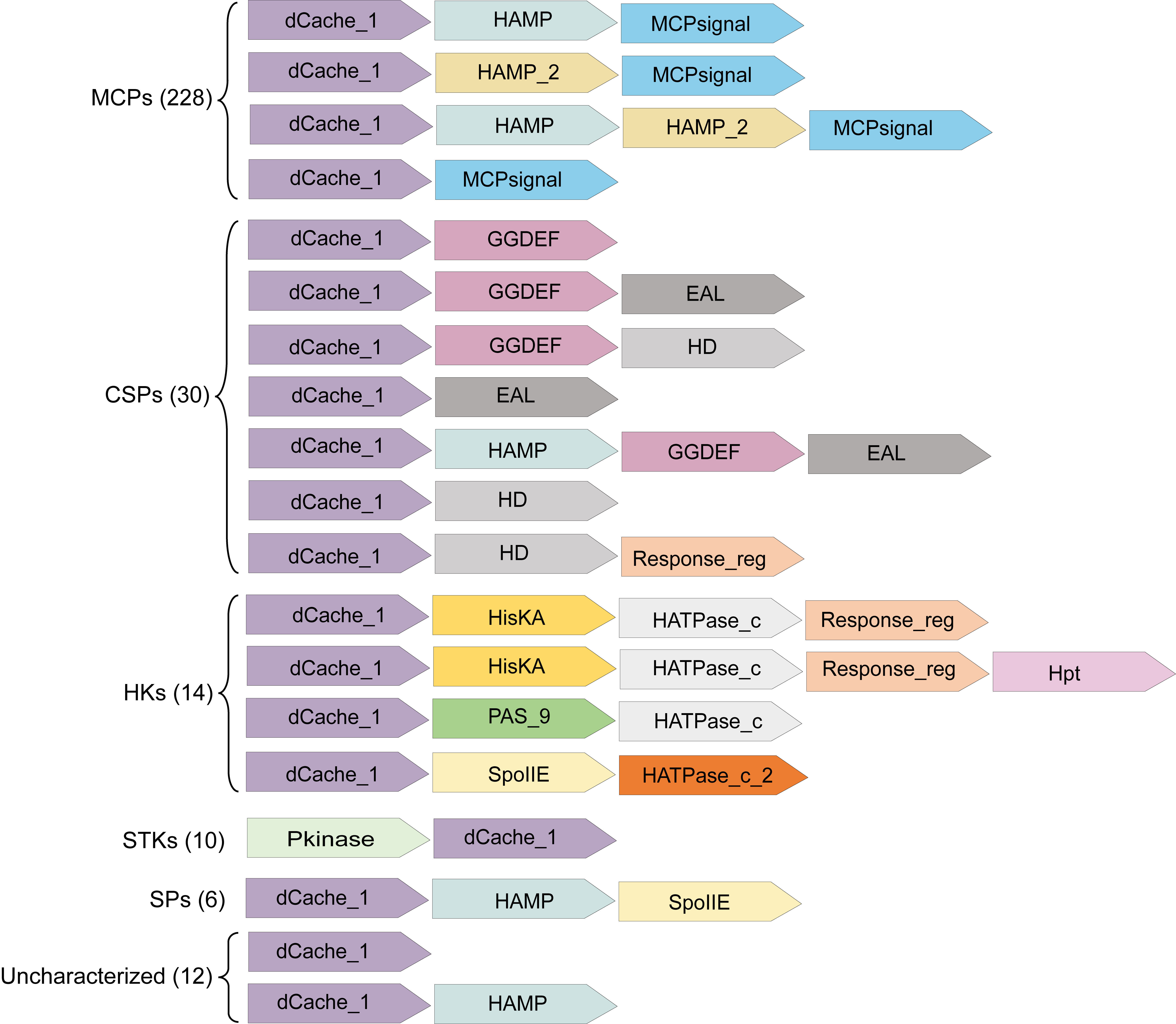


**Figure S4.** The domain architecture and functional modules of 300 dCache_1-type AI-2 receptors in human gut microbiota. 228 are MCPs, 30 are CSPs, 14 are HKs, 10 are STKs, 6 are SPs, and 12 are uncharacterized proteins without any predicted functional domains or motifs.

**
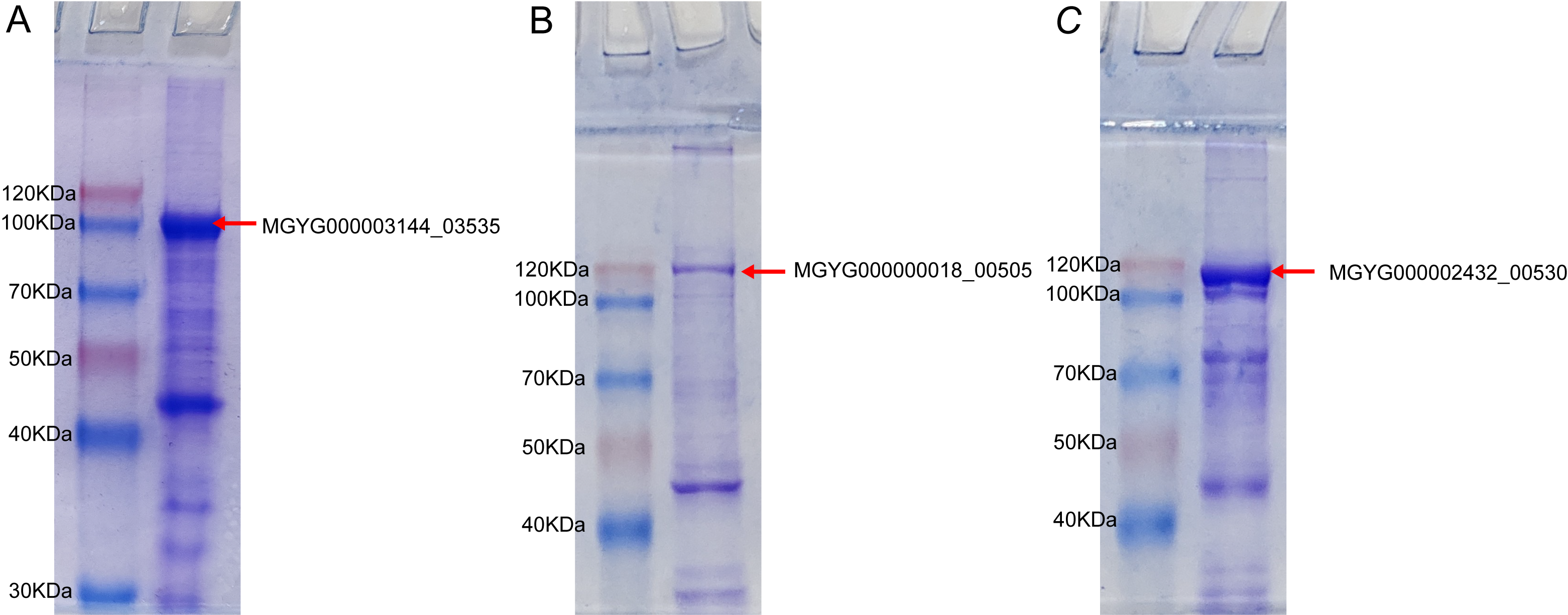
**

**Figure S5.** SDS-PAGE gels of three purified full-length dCache_1-type AI-2 receptors. After inverted membrane extraction and further purification by Ni^2+^-NTA affinity chromatography, full-length MGYG000003144_03535 (A), MGYG000000018_00505 (B), and MGYG000002432_00530 (C) were subjected to SDS-PAGE analysis to examine the purity.

**
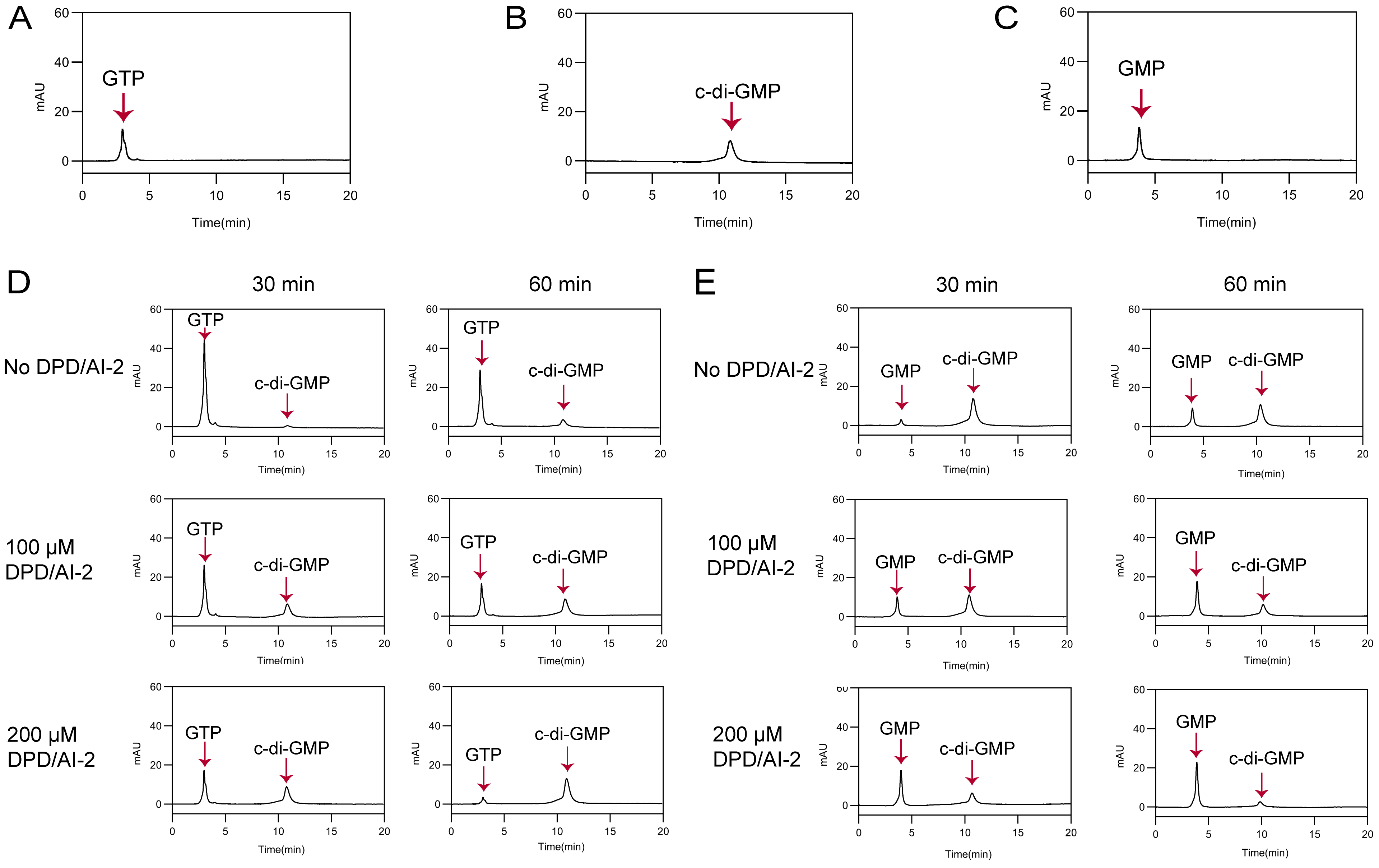
Figure S6.** AI-2 induces the activities of MGYG000003144_03535 and MGYG000000018_00505 in c-di-GMP synthesis and degradation, respectively. **A.** The HPLC profile of GTP (30 μM). **B.** The HPLC profile of c-di-GMP (30 μM). **C.** The HPLC profile of GMP (30 μM). **D.** AI-2 induces the activity of MGYG000003144_03535 in c-di-GMP synthesis. MGYG000003144_03535 was incubated with GTP in the absence and presence of DPD/AI-2 (0, 100, and 200 μM) at 30°C for 30 and 60 min. The products were analyzed by HPLC. **E.** AI-2 induces the activity of MGYG000000018_00505 in c-di-GMP degradation. MGYG000000018_00505 was incubated with c-di-GMP in the absence and presence of DPD/AI-2 (0, 100, and 200 μM) at 30°C for 30 and 60 min. The products were analyzed by HPLC. HPLC spectra shown are representatives of three independent experiments with similar results.

**
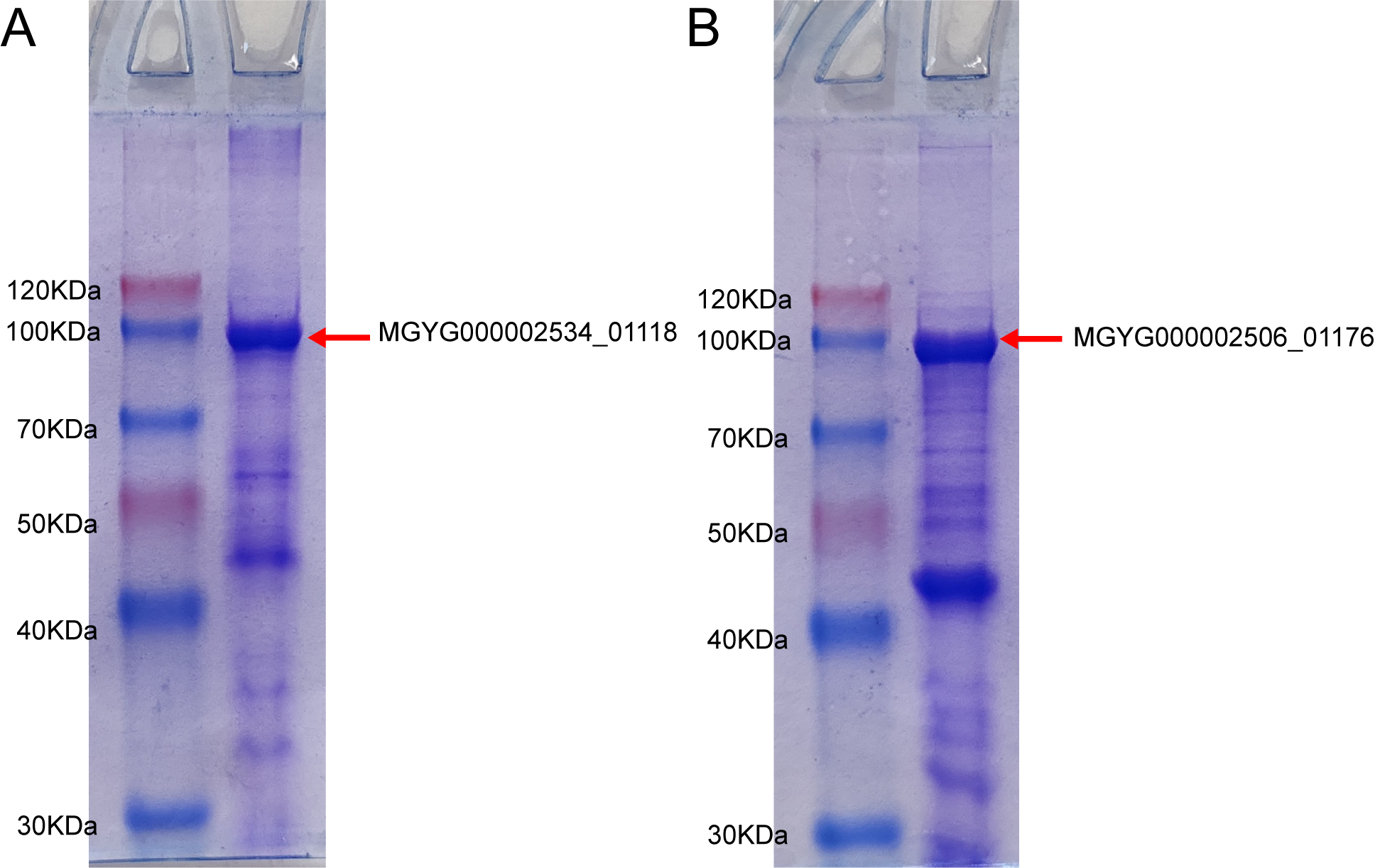
**

**Figure S7.** SDS-PAGE gels of two purified full-length GAPES1-type AI-2 receptors. After inverted membrane extraction and further purification by Ni^2+^-NTA affinity chromatography, full-length MGYG000002534_01118 (A) and MGYG000002506_01176 (B) were subjected to SDS-PAGE analysis to examine the purity.

**
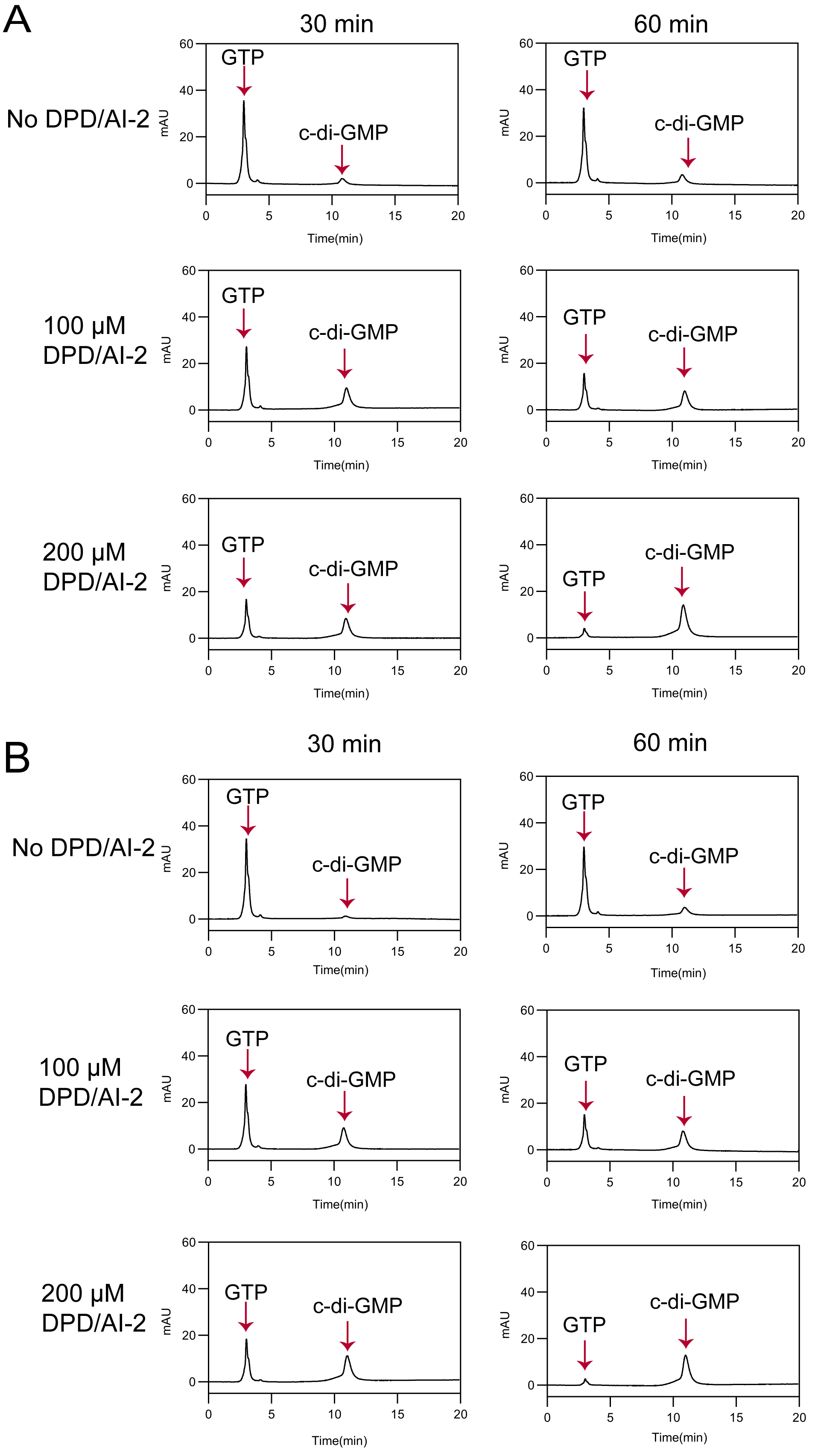
**

**Figure S8.** AI-2 induces the activities of MGYG000002534_01118 and MGYG000002506_01176 in c-di-GMP synthesis. MGYG000002534_01118 (A) and MGYG000002506_01176 (B) were incubated with GTP in the absence and presence of DPD/AI-2 (0, 100, and 200 μM) at 30°C for 30 and 60 min. The products were analyzed by HPLC. HPLC spectra shown are representatives of three independent experiments with similar results. Overall distribution profiles of AI-2 receptors within the human gut microbiota.


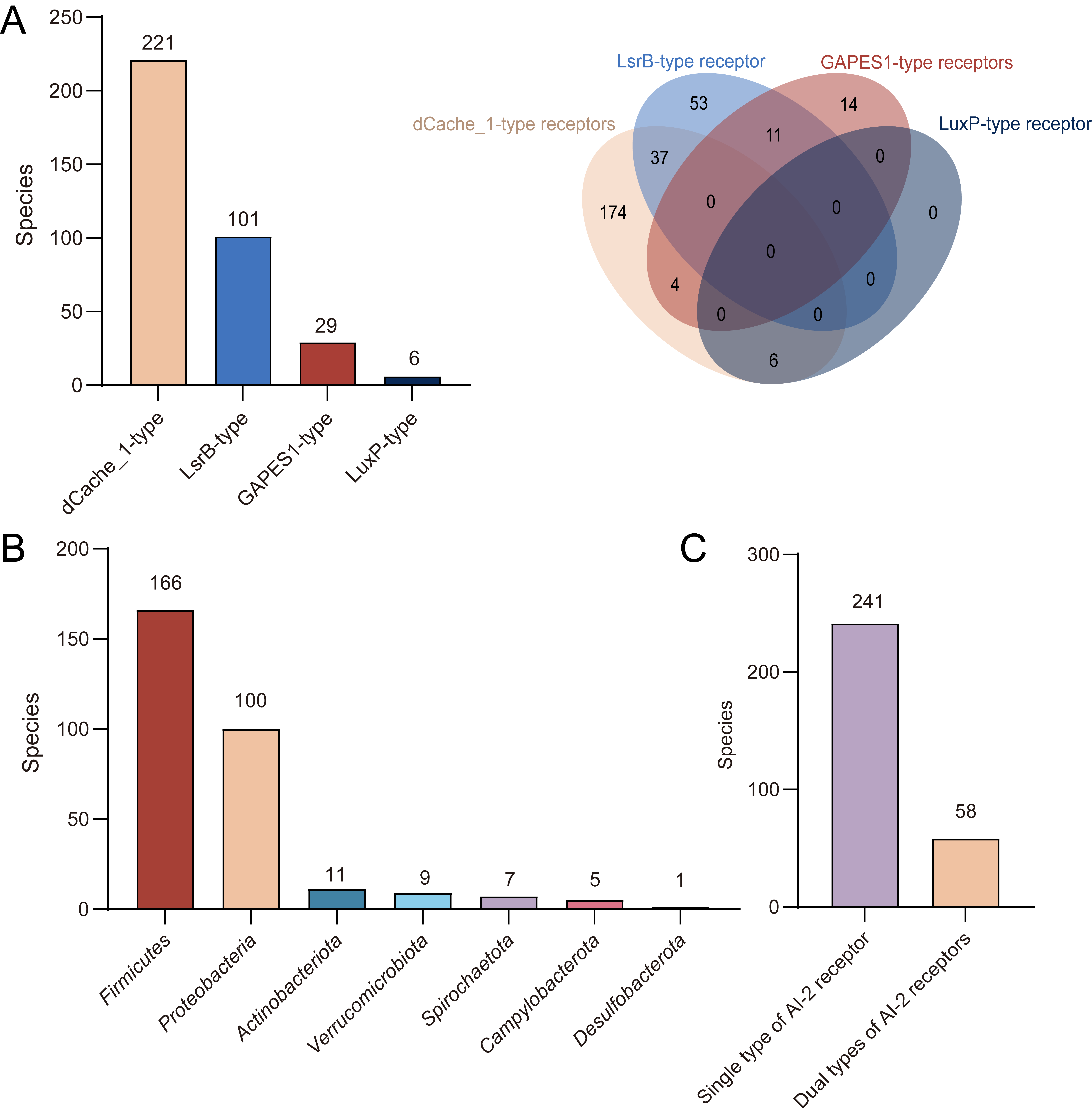


**Figure S9. A.** Venn diagram showing numbers of bacterial species possessing four types of AI-2 receptors. Ellipses with different colors represent different types of AI-2 receptors. The numbers in the overlapping areas of different colors denote the number of species containing multiple types of receptors. **B.** The phylum-level distribution of bacterial species containing AI-2 receptors. **C.** 241 species have single type of AI-2 receptors, and 58 species harbor dual types of AI-2 receptors.


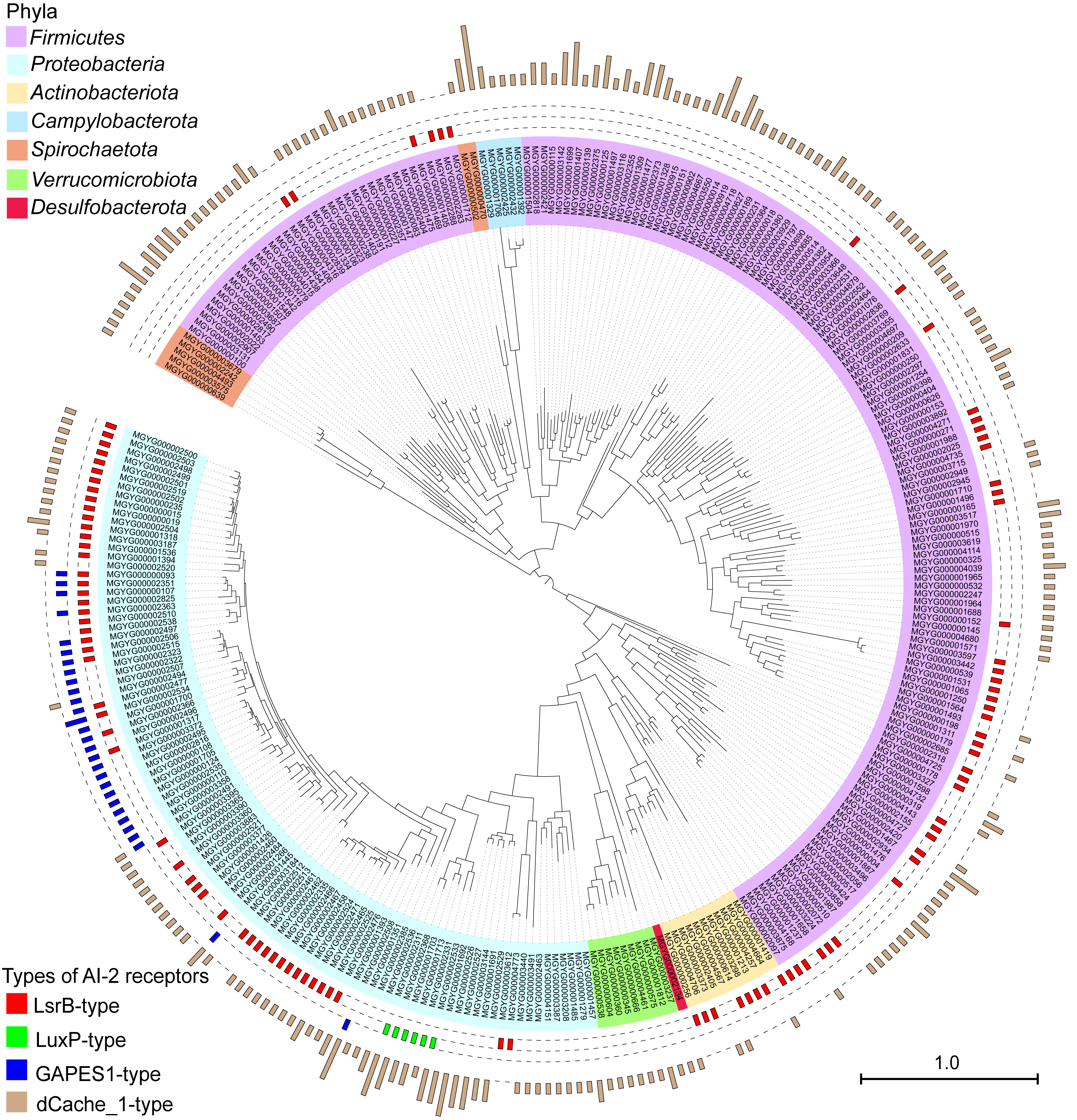


**Figure S10.** Phylogenetic analysis of 299 species possessing AI-2 receptors within human gut microbiota. Bars in the outer four layers represent the distribution and number of dCache_1-, GAPES1-, LuxP-, and LsrB-type AI-2 receptors present in the corresponding species, with their existence depicted in brown, blue, green, and red, respectively. This phylogenetic tree was constructed based on the genomes of 299 species possessing AI-2 receptors using FastTree software (v2.1.11). iTOL (v5) were used to visualize it. The scale bar represents 1.0 amino acid substitutions per site.

**
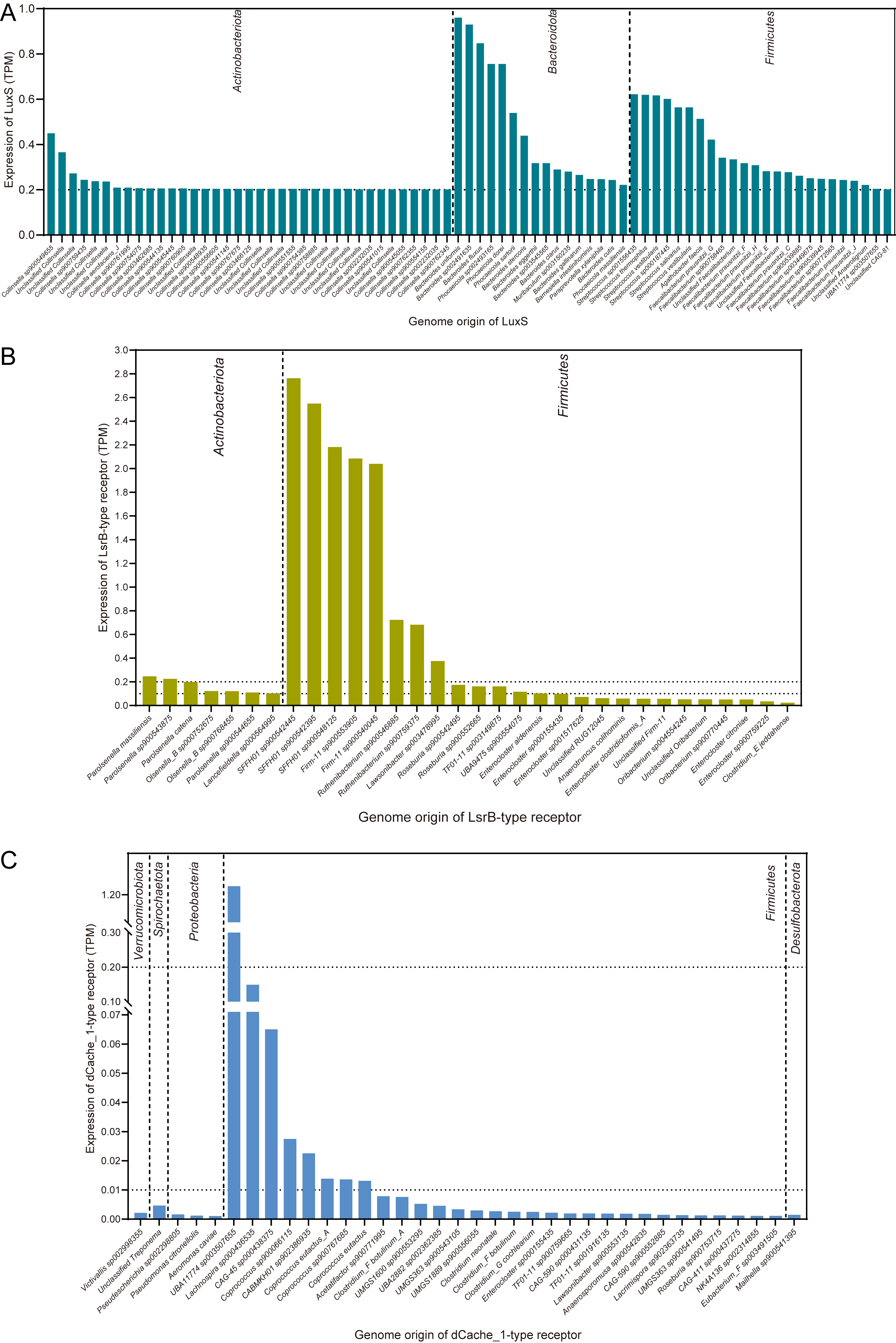
**

**Figure S11.** Expression of the AI-2 synthase LuxS, and the LsrB-, and dCache_1-type AI-2 receptors within the healthy human gut metatranscriptome datasets. **A**. Species expressing *luxS* with a TPM value over 0.2. A total of 77 species were distributed in *Actinobacteriota* (37 species), *Firmicutes* (24 species), and *Bacteroidetes* (16 species), respectively. **B**. The top 32 species with relatively high TPM values (TPM > 0.02) among the 41 species expressing LsrB-type AI-2 receptors in the healthy human gut metatranscriptome data. **C**. The top 36 species with relatively high TPM values (TPM > 0.001) among the 63 species expressing dCache_1-type AI-2 receptors in the healthy human gut metatranscriptome data.


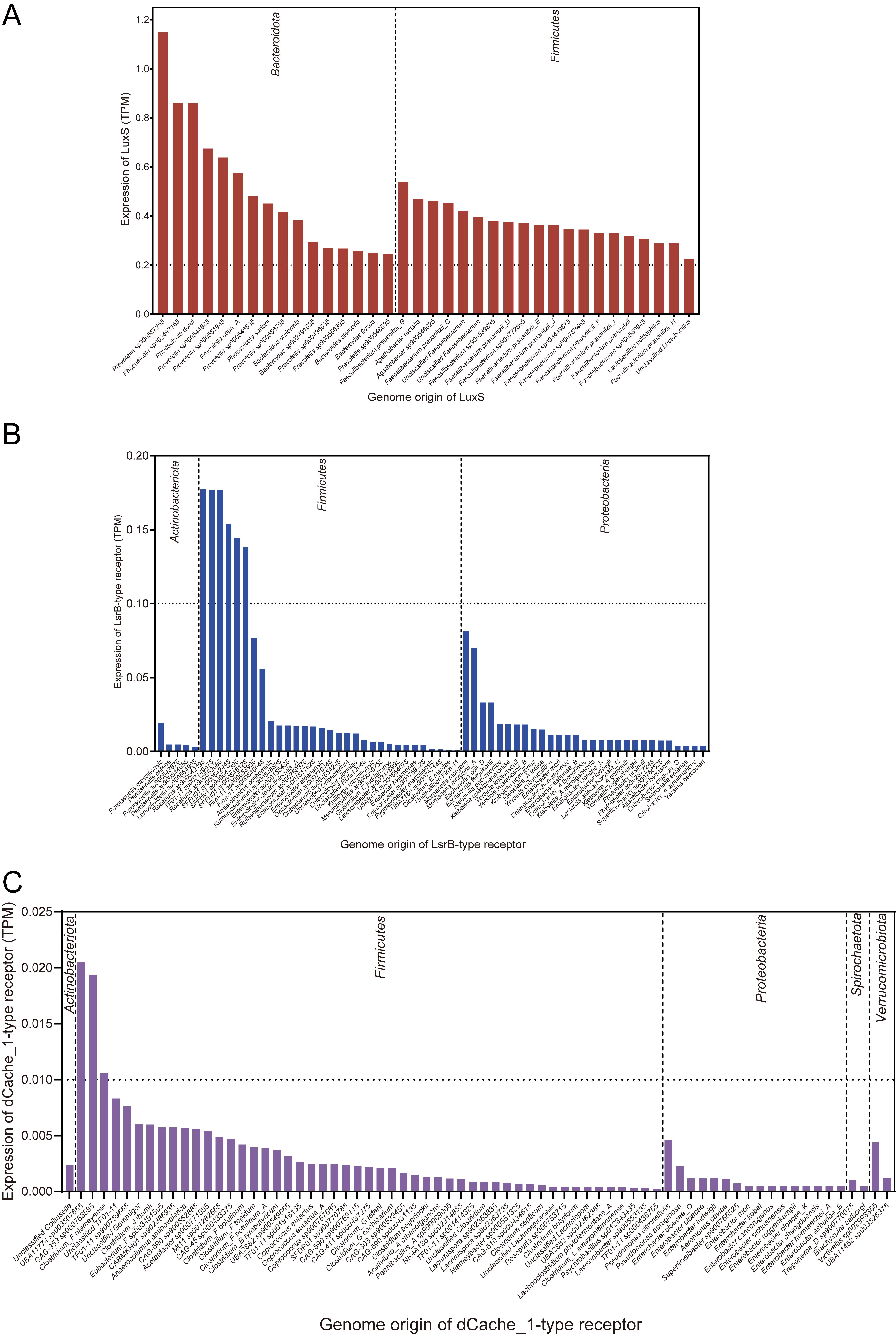


**Figure S12.** Expression of the AI-2 synthase LuxS, LsrB-type, and dCache_1-type AI-2 receptors within the unhealthy human gut metatranscriptome datasets. **A**. Species expressing *luxS* with a TPM value over 0.2. A total of 36 species were distributed in *Bacteroidetes* (16 species) and *Firmicutes* (20 species), respectively. **B**. TPM values of all expressed LsrB-type AI-2 receptors within the unhealthy human gut metatranscriptome datasets. 65 species expressing *lsrB* were distributed in *Firmicutes* (31 species), *Actinobacteria* (5 species), and *Proteobacteria* (29 species). **C**. TPM values of all expressed dCache_1-type AI-2 receptors within the unhealthy human gut metatranscriptome datasets. 72 species expressing genes encoding dCache_1-type AI-2 receptors were distributed in *Firmicutes* (51 species), *Proteobacteria* (16 species), *Verrucomicrobiota* (2 species), *Spirochaetota* (2 species), and *Actinobacteriota* (1 species).

**Supplementary References**

1. Franzosa EA, Morgan XC, Segata N et al. Relating the metatranscriptome and metagenome of the human gut. *Proc Natl Acad Sci U S A* 2014;**111**:E2329-38. https://doi.org/10.1073/pnas.1319284111

2. Lloyd-Price J, Arze C, Ananthakrishnan AN et al. Multi-omics of the gut microbial ecosystem in inflammatory bowel diseases. *Nature* 2019;**569**:655-62. https://doi.org/10.1038/s41586-019-1237-9

3. Ewels P, Magnusson M, Lundin S et al*.* MultiQC: summarize analysis results for multiple tools and samples in a single report. *Bioinformatics* 2016;**32**:3047-8. https://doi.org/10.1093/bioinformatics/btw354

4. Bolger AM, Lohse M, Usadel B. Trimmomatic: A flexible trimmer for illumina sequence data. *Bioinformatics* 2014;**30**:2114-20. https://doi.org/10.1093/bioinformatics/btu170

5. Langmead B, Salzberg SL. Fast gapped-read alignment with Bowtie 2. *Nat Methods* 2012;**9**:357-9. https://doi.org/10.1038/nmeth.1923

6. Li H, Handsaker B, Wysoker A et al. The Sequence Alignment/Map format and SAMtools. *Bioinformatics* 2009;**25**:2078-9. https://doi.org/10.1093/bioinformatics/btp352

7. Liao Y, Smyth GK, Shi W. FeatureCounts: An efficient general purpose program for assigning sequence reads to genomic features. *Bioinformatics* 2014;**30**:923-30. https://doi.org/10.1093/bioinformatics/btt656

8. Zhang L, Li S, Liu X et al. Sensing of autoinducer-2 by functionally distinct receptors in prokaryotes. *Nat Commun* 2020;**11**:5371. https://doi.org/10.1038/s41467-020-19243-5

9. Li S, Sun H, Li J et al*.* Autoinducer-2 and bile salts induce c-di-GMP synthesis to repress the T3SS via a T3SS chaperone. *Nat Commun* 2022;**13**:6684. https://doi.org/10.1038/s41467-022-34607-9

10. Zhang H, Zhao W, Yang W et al. Autoinducer-2 enhances the defense of *Vibrio furnissii* against oxidative stress and DNA damage by modulation of c-di-GMP signaling via a two-component system. *mBio* 2025:e0292224. https://doi.org/10.1128/mbio.02922-24
